# Neddylation of Coro1a determines the fate of multivesicular bodies and biogenesis of extracellular vesicles

**DOI:** 10.1002/jev2.12153

**Published:** 2021-10-08

**Authors:** Xuefeng Fei, Zhijie Li, Diya Yang, Xianghui Kong, Xinliang Lu, Yingying Shen, Xu Li, Shaofang Xie, Jiaoli Wang, Yongchao Zhao, Yi Sun, Jing Zhang, Zhaoming Ye, Jianli Wang, Zhijian Cai

**Affiliations:** ^1^ Institute of Immunology Department of Orthopaedics of the Second Affiliated Hospital Zhejiang University School of Medicine Hangzhou China; ^2^ Xinyuan Institute of Medicine and Biotechnology School of Life Sciences Zhejiang Sci‐Tech University Hangzhou China; ^3^ School of Life Science Westlake University Hangzhou China; ^4^ Key Laboratory of Clinical Cancer Pharmacology and Toxicology Research of Zhejiang Province Affiliated Hangzhou First People's Hospital, Zhejiang University School of Medicine Hangzhou China; ^5^ Zhejiang University Cancer Centre Hangzhou China; ^6^ Cancer Institute of the Second Affiliated Hospital, and Institute of Translational Medicine Zhejiang University School of Medicine Hangzhou China; ^7^ Department of Pathology of the First Affiliated Hospital Zhejiang University School of Medicine Hangzhou China; ^8^ Department of Orthopaedics Musculoskeletal Tumour Centre of the Second Affiliated Hospital Zhejiang University School of Medicine Hangzhou China; ^9^ Institute of Immunology Bone Marrow Transplantation Centre of the First Affiliated Hospital Zhejiang University School of Medicine Hangzhou China; ^10^ Institute of Haematology Zhejiang University & Zhejiang Engineering Laboratory for Stem Cell and Immunotherapy Hangzhou China

**Keywords:** Coro1a, extracellular vesicles, multivesicular bodies, neddylation, Rab7

## Abstract

Multivesicular bodies (MVBs) fuse with not only the plasma membranes to release extracellular vesicles (EVs) but also lysosomes for degradation. Rab7 participates in the lysosomal targeting of MVBs. However, the proteins on MVB that directly bind Rab7, causing MVB recruitment of Rab7 remain unidentified. Here, we show that Coro1a undergoes neddylation modification at K233 by TRIM4. Neddylated Coro1a is associated with the MVB membrane and facilitates MVB recruitment and activation of Rab7 by directly binding Rab7. Subsequently, MVBs are targeted to lysosomes for degradation in a Rab7‐dependent manner, leading to reduced EV secretion. Furthermore, a decrease in neddylated Coro1a enhances the production of tumour EVs, thereby promoting tumour progression, indicating that neddylated Coro1a is an ideal target for the regulation of EV biogenesis. Altogether, our data identify a novel substrate of neddylation and reveal an unknown mechanism for MVB recruitment of Rab7, thus providing new insight into the regulation of EV biogenesis.

## 1 INTRODUCTION

Extracellular vesicles (EVs) are mainly classified into two major groups based on their size and origin, termed ectosomes and exosomes. Ectosomes are vesicles directly shedding from plasma membrane (PM), which generates microvesicles, microparticles and large vesicles with diameter ranging from 50 nm to 1 μm. Exosomes are vesicles 30–150 nm in diameter produced by endosome pathway (Kalluri & LeBleu, 2020). EVs contain proteins, RNA, DNA, lipids and metabolites from their parental cells. When taken up by recipient cells, EVs can influence the functions of these cells through transferring their contents (Colombo et al., 2014). EVs are involved in the progression of diverse diseases (Castaño et al., 2019; Hough & Deshane, 2019; Kalluri & LeBleu, 2020). Therefore, targeting EV biogenesis is a potential strategy for the treatment of the related diseases, which urges an improved understanding of EV biogenesis. During the biogenesis of EV subset exosomes, invagination of the PM forms early endosomes (EEs). Then, EEs mature into late endosomes (LEs) and eventually multivesicular bodies (MVBs) through additional inward invagination of the endosomal limiting membrane. MVBs, which contain many intraluminal vesicles (ILVs), can fuse with either lysosomes to be degraded or the PM to release the ILVs as exosomes (Kalluri & LeBleu, 2020). Therefore, the fate of MVBs determines the biogenesis of EVs. However, the mechanisms responsible for the fusion of MVBs with the lysosome or PM are still largely unknown.

Ubiquitin and ubiquitin‐like modifications contribute to the regulation EV biogenesis. The E3 ubiquitin ligase Nedd4 bind the Nedd4 family‐interacting protein Ndfip1 and mediate the ubiquitination of substrate proteins. Ndfip1 can promote the secretion of EVs, resulting in an increase in ubiquitinated proteins in secreted EVs (Putz et al., 2008). The modification of proteins by the ubiquitin‐like process ISGylation inhibits EV secretion by promoting the lysosomal‐mediated degradation of MVBs (Villarroya‐Beltri et al., 2016). Neddylation refers to the covalent attachment of the ubiquitin‐like protein NEDD8 to a target protein, which is related to neurodegenerative diseases, cancer and infection (He et al., 2009; Li et al., 2021). Analogous to ubiquitin, NEDD8 covalently bind lysine residues in the substrate via G76. UBA3 and NAE1 form a heterodimer that functions as a NEDD8 E1‐activating enzyme, mediating activation and transfer of NEDD8 to the cysteine active site of the E2 conjugating enzyme UBE2M or UBE2F. Finally, under E3 ligase catalysis, NEDD8 forms an isopeptide bond with the lysine residue of the substrate (Zhao et al., 2014). Whether neddylation modification can also regulate EV biogenesis has yet to be explored.

Here, we show that neddylation has a negative effect on EV biogenesis. We identified Coro1a as a new neddylation substrate. Coro1a is neddylated by TRIM4 E3 ligase at K233. Neddylated Coro1a (NEDD8‐Coro1a) then directly binds and recruits Rab7 to MVBs, which promotes lysosomal degradation of the MVBs, leading to reduced fusion of MVBs to the PM and EV secretion. Furthermore, we confirmed that Coro1a is an effective target for the regulation of EVs when exemplified in the mouse tumour model. These findings demonstrate that NEDD8‐Coro1a determines the lysosomal fusion of MVBs by recruiting Rab7 and thus reveal a novel mechanism by which EV biogenesis is regulated.

## 2 MATERIALS AND METHODS

### 2.1 Reagents

The information of all the reagents used in this article were listed in Table S1.

### 2.2 Mice

C57BL/6J mice (female, 6–8 weeks old) were purchased from the Shanghai SLAC Laboratory Animal Co., Ltd. (Shanghai, China). *Coro1a^fl/fl^* mice were constructed by Cyagen Biosciences (Suzhou, Jiangsu, China), and *Lysm^cre^* mice were provided by the same company. *Ube2f^fl/fl^* and *Ube2m^fl/fl^* mice were generated as described (Wu et al., 2020). The mice were housed in a specific pathogen‐free facility, and the experimental protocols were approved by the Animal Care and Use Committee of the School of Medicine at Zhejiang University. Mouse identification primer sequences were listed in Table S1.

### 2.3 Cells

HEK293 and HeLa cells were obtained from the Chinese Academy of Sciences (Shanghai, China). RAW264.7 and MC38 cells were purchased from the American Type Culture Collection (ATCC). The cells were cultured in Dulbecco's modified Eagle's medium (DMEM) supplemented with 10% (v/v) fetal bovine serum (FBS) and 100 μg ml^−1^ penicillin‐streptomycin. To generate bone marrow‐derived macrophages (BMDMs), tibias and femurs were removed with sterile techniques, and the bone marrow was flushed with fresh RPMI‐1640 medium supplemented with 10% (v/v) FBS (HyClone) and 100 μg ml^−1^ penicillin‐streptomycin, then the bone marrow cells were plated in medium supplemented with GM‐CSF (10 ng ml^−1^) for 6 days for next experiments. All cells were maintained at 37°C in a humidified atmosphere containing 5% CO_2_.

### 2.4 Plasmids

The cDNA sequences for human CD63 (hCD63) tagged with HA, hCoro1a tagged with Flag or HA, and hRab7a tagged with His and fused with mCherry tag at their N‐termini were cloned by PCR from the cDNA of HEK293 cells and inserted into the lentiviral overexpression plasmid plvx‐mCherry‐C1 backbone (TaKaRa). hNEDD8 tagged with HA, hCoro1a tagged with Flag and hTRIM21, hNAE1, hUBA3, hUBE2F, and hUBE2M tagged with Myc were cloned by PCR from the cDNA of HEK293 cells and inserted into the pcDNA3.1 backbone (Invitrogen). pcDNA3.1‐hCoro1a‐K233R‐Flag, mCherry‐hCoro1a‐K233R‐Flag, mCherry‐hRab7a^Q67L^‐His and mCherry‐hRab7a^T22N^‐His were cloned from the WT plasmids by base mutation. mCherry‐hTRIM4‐His, mCherry‐mouse Coro1a (mCoro1a)‐Flag, mCherry‐mCoro1a‐K233R‐Flag and mCherry‐CD63‐GFP were obtained from the MiaoLing Plasmid Sharing Platform (Wuhan, Hubei, China). Rab5^Q79L^‐GFP was provided by Prof. Yuehai Ke (Zhejiang University, Hangzhou, Zhejiang, China). All the constructs described above were fully verified by sequencing.

### 2.5 DNA and siRNA transfection

HEK293, HeLa and MC38 cells were transfected with plasmids using JetPEI Transfection Reagent (Polyplus) or PEI (Polysciences) according to the manufacturer's protocol.

HEK293 and HeLa cells were transfected with scramble NC or targeted siRNA using INTERFERin (Polyplus) or TransIT‐TKO Transfection Reagent (Mirus Bio) according to the manufacturer's instructions. The sequences of siRNAs were listed in Table S1.

### 2.6 Cellular treatments

To inhibit neddylation, cells were cultured with 100 nM MLN4924 (Selleck) for 12 h. To inhibit lysosome function, cells were cultured with 20 nM Baf A1(MCE) for 12 h. To inhibit cullin3 neddylation, cells were cultured with 10 μM DI‐591DD or DI‐591, both of which were kindly provided by Prof. Shaomeng Wang (University of Michigan, Ann Arbor, MI, USA), for 12 h.

### 2.7 EV isolation

FBS was ultracentrifugated at 120,000 × *g* for 10 h to remove EVs and then added into DMEM at final concentration of 10% (v/v). HEK293 and MC38 cells were cultured in this DMEM until approximate 90% confluence in 10‐cm cell culture dishes. Then, the conditioned media were collected and subjected to a centrifugation step at 300 × *g* and room temperature (RT) for 10 min to pellet and remove the cells. All subsequent centrifugation steps were performed at 4°C. Next, the supernatant was spun at 2,000 × *g* for 20 min to remove debris and apoptotic bodies and then centrifuged at 10,000 × *g* for 30 min to remove large EVs. Then, the media supernatants from the centrifugation step at 10,000 × *g* were passed through 0.22 μm PVDF filters (Millipore) and subjected to ultracentrifugation at 120,000 × *g* for 70 min by Beckman XPN‐100 with an SW 32 Ti swinging bucket rotor (Beckman Coulter) to sediment EVs. The crude EV pellets were resuspended in a large volume of ice‐cold PBS, followed by ultracentrifugation at 120,000 × *g* for 70 min to wash the samples. The final pellets were resuspended in ice‐cold PBS. The concentrations of exosomal proteins were measured using a BCA Protein Assay Kit (Thermo Fisher) according to the manufacturer's instructions.

### 2.8 Semiquantitative EV assay

To detect EVs in the cell culture supernatants, supernatants containing FBS depleted of bovine serum EVs were cleared by centrifugation at 300 × *g* for 10 min and 2,000 × *g* for 20 min. Four‐micrometre aldehyde sulphate beads (Invitrogen) were first coated with purified anti‐CD63 antibodies (BioLegend) and then blocked with FBS depleted of bovine serum EVs at RT for 1 h. The beads were washed twice in PBS and centrifuged at 3000 × *g* for 5 min. The cleared supernatants (1 ml) were incubated with anti‐CD63‐coupled beads overnight at 4°C with shaking. The beads were washed twice in PBS and incubated with anti‐CD9 or anti‐CD81 antibodies (BioLegend) for 30 min at 4°C. After washing twice in PBS, the beads were acquired on an ACEA NovoCyte and the data were analysed with NovoExpress software (ACEA Biosciences). The negative staining threshold was obtained from beads incubated with unconditioned medium.

To detect CD9 or CD81 on EVs, 5 μg of EVs were adsorbed by 4‐μm aldehyde sulphate beads for 20 min and then blocked with EV‐free FBS at RT for 30 min. Then, the beads were washed twice in PBS by centrifuged at 3000 × *g* for 5 min and incubated with anti‐CD9 or anti‐CD81 antibodies for 30 min at 4°C. To detect Coro1a on CD63^+^ EVs, 5 μg of EVs were captured by anti‐CD63‐coupled beads and stained with anti‐Coro1a antibodies (Abcam) for 30 min at 4°C followed by DyLight 488 IgG (MultiSciences) incubation for another 30 min at 4°C. To detect CD63 or Annexin A1 on Coro1a^+^ EVs, 5 μg of EVs were captured by anti‐Coro1a‐coupled beads and stained with anti‐CD63 or anti‐Annexin A1 antibodies (BioLegend). After washed twice in PBS, the beads were acquired on an ACEA NovoCyte, and the data were analysed with NovoExpress software. The negative staining threshold was obtained with the corresponding IgG isotype control (Abclonal; BioLegend).

### 2.9 Nanoparticle tracking analysis

To measure particle size and concentration, EVs were analysed by nanoparticle tracking analysis (NTA) using a NanoSight NS300 system (Malvern PANalytical) configured with a 488 nm laser and high‐sensitivity sCMOS camera and finally analysed with NTA 3.2 or 3.3 software.

### 2.10 Iodixanol density gradient fractionation

Iodixanol density gradient medium (StemCell) was prepared in ice‐cold PBS immediately before use to generate a discontinuous step gradient (12–28%). The 28% iodixanol solution was added to the bottom of a centrifugation tube, and of iodixanol at decreasing concentrations in PBS were carefully layered on top of the iodixanol solution, yielding the complete gradient. Identical gradients without sample were generated and ultracentrifugated by the same way for later determination of fraction densities. The density of fractions was measured by refractometry. Crude EV pellets resuspended in ice‐cold PBS were added to the top of the centrifugation tube. The gradient was subjected to ultracentrifugation at 120,000 × *g* for 16 h at 4°C using Beckman XPN‐100 with an SW 41 Ti swinging bucket rotor. Nine individual 1‐ml fractions were collected from the top of the gradient. For WB analysis, each individual 1 ml fraction was transferred to a new ultracentrifugation tube, diluted in PBS and subjected to ultracentrifugation at 120,000 × *g* for 4 h at 4°C using an SW 32 Ti swinging bucket rotor. The resulting pellets were lysed in 5 × SDS buffer on ice and boiled for 10 min at 100°C.

### 2.11 Electron microscope

For negative EV staining, 200‐mesh carbon films were hydrophilized with a glow discharge instrument at 15 mA for 25 s. The EV solution was pipetted on the 200‐mesh carbon‐coated copper grids and kept at RT for 1 min. After the excess suspension had been removed with filter paper and the grid had been washed twice with ddH_2_O, the EVs were negatively stained with 2% uranyl acetate at RT for 1 min, and the excess suspension was removed and dried naturally. Images were acquired by electron microscope (EM) (Tecnai G2 Spirit 120 kV, Thermo FEI).

For MVB analysis, HEK293 cells were placed in 2.5% glutaraldehyde in PBS and fixed overnight at 4°C. The samples were washed three times in PBS for 10 min each and then fixed in 1% osmium tetroxide for 60 min at RT. The samples were stained in 2% uranium acetate for 30 min after being washed three times in PBS. Then, the samples were dehydrated through a graded ethanol series (50%, 70%, 90% and 100%) and 100% acetone. Next, the samples were embedded in a 1:1 solution of epon:acetone for 2 h at RT and a 3:1 solution of epon:acetone overnight at RT. The next day, they were placed in fresh epon for 8 h at 37°C and then embedded in epon for 72 h at 65°C. Thin sections were cut on an ultramicrotome (UC7, Leica), collected on grids and examined by EM (Tecnai G2 Spirit 120 kV).

For ultrathin cryosectioning and immunogold labelling, HEK293 cells were fixed in 4% paraformaldehyde with 0.5% glutaraldehyde in 0.1 M PBS (pH 7.2–7.4) at 37°C for 1 h. The fixed samples were washed and incubated in 20% gelatin at 37°C for 10 min and then placed on ice for 30 min. The samples were incubated in a 2.3 M sucrose solution with rotation at RT overnight, cut into blocks at ‐80°C and further cut into 100 nm ultrathin sections at ‐120°C. After being washed six times in 0.1 M PBS and blocked with 0.1% BSA for 15 min at RT, the samples were incubated with the primary antibodies (Rab7, Bioss; Coro1a, Abcam) for 2 h at RT, followed by incubation with the second antibodies: AffiniPure goat anti‐rabbit IgG (H+L) conjugated with 18 nm colloidal gold (EM grade, 1:20). After thorough washing, the samples were stained with 0.3% uranyl acetate for 5 min, the excess liquid was removed, and the samples were dried naturally. Finally, the samples were examined by EM (Tecnai G2 Spirit 120 kV).

### 2.12 ELISA

Serum EVs in the individual samples were measured by sandwich ELISA. Briefly, 96‐well ELISA plates were coated with 4 μg ml^−1^ purified anti‐mouse CD63 antibodies (BioLegend) in coating buffer and incubated overnight at 37°C. After blocking with assay diluent, serum samples in triplicate were added to individual wells and incubated overnight at 37°C. The plates were washed, and the bound EVs were detected by incubation with biotin anti‐mouse CD9, biotin anti‐mouse CD81 or biotin anti‐mouse F4/80 antibodies (4 μg ml^−1^) (BioLegend) and avidin‐HRP for 2 h at 37°C. Then, the signal was developed with TMB, and the samples were blocked with 2 M H_2_SO_4_, after which absorbance at 450 nm was measured with a SpetraMax M5 microplate reader (Molecular Devices).

### 2.13 Immunofluorescence staining and confocal microscopy

HeLa cells were cultured overnight on glass coverslips and then treated with inhibitors or subjected to transfection. After being washed three times with PBS, the cells were fixed with ‐20°C prechilled methyl alcohol for 10 min and permeabilized with 0.1% Triton X‐100. After blocking with 5% BSA and 3% goat serum in PBS, the cells were incubated with primary antibodies (CD63, Invitrogen; HRS, Abcam; EEA1, abcam; LAMP1, abcam; Flotillin‐1, Abcam) overnight at 4°C in blocking buffer. The following day, after three washes in PBS, the cells were incubated with secondary antibodies (DyLight 488, DyLight 594, MultiSciences) for 30 min at RT, washed in PBS, and then mounted in antifade mounting medium with DAPI. The samples were imaged using an Olympus IX83‐FV3000 confocal microscope (Olympus). Images were analysed with ImageJ software.

### 2.14 The proximity ligation assay

The proximity ligation assay (PLA) were carried out using Duolink In Situ PLA reagents according to the manufacturer's instructions (Sigma) by using the primary antibodies (Coro1a, Abcam; Rab7, Abcam; GDI2, Abclonal; RILP, Proteintech). Samples were imaged using an Olympus IX83‐FV3000 confocal microscope (Olympus). Images were analysed with ImageJ software.

### 2.15 Total internal reflection fluorescence microscopy

HeLa cells were transfected with the mCherry‐CD63‐HA plasmid for 36 h, and then the cells were incubated in PBS before analysis by total internal reflection fluorescence (TIRF) microscopy. For TIRF microscopy with an Olympus IX83 microscope (Olympus), the penetration depth (δ) of the evanescent field used to excite the fluorophore was set to 150 nm. Frames were acquired at 10 Hz in stacks of 400 images with an exposure time of 100 ms. Fluorescence image acquisitions were collected with CellSens software (Olympus). mCherry^+^ CD63 vesicles were quantified using ImageJ software. To analyse vesicle motion, time‐series images were captured with a Nikon N‐STORM & A1 Cell TIRF system using a DU897 EMCCD 100 × oil TIRF objective and the fluorescence image acquisitions were collected with Nis‐Elements software (Nikon). The mean square displacement was calculated with Imaris 9.5 software (Bitplane AG). The diffusion coefficient, which was the slope of the linear fit of the first 15 points of the mean square displacement curve, was then calculated as *D_xy_* = s/4.

### 2.16 Three‐dimensional structured illumination microscopy

Sample preparation was carried out as described above with primary antibodies (Coro1a, Abcam; CD63, Invitrogen) and secondary antibodies (Alexa Fluor 568, Abcam; DyLight 488, MultiSciences). Three‐dimensional structured illumination microscopy (3D‐SIM) images were captured with a Nikon‐SIM equipped with an ECLIPSE Ti, and a CFI Apochromat TIRF 100 × H objective lens and recorded as vertical z stacks. The images were then processed by using NIS‐Elements AR (Nikon) for three‐dimensional reconstruction and were analysed by Imaris 9.5 to generate surface renderings and calculate the contact surface area.

### 2.17 Immunoprecipitation and western blotting

For western blotting (WB) assay, total cells and EVs were washed with ice‐cold PBS and lysed in SDS buffer on ice and boiled for 10 min at 100°C. Then, the samples were resolved by SDS‐PAGE followed by transfer onto PVDF membranes (Millipore) and probed with the indicated primary antibodies (CD63, Abclonal; Alix, Proteintech; Tsg101, Abclonal; CD81, Affinity; Coro1a, Abcam; GM130, Abcam; NEDD8, Abcam; Ub, CST; UBA3, Abcam; Cullin3, Sangon Biotech; UBE2F, Bioss; UBE2M, Abcam; TRIM4, Abclonal; Monla, Abclonal; Monlb, Abclonal; Rab7, Abcam; β‐Actin, Abclonal) and second antibodies (Goat anti‐mouse IgG HRP, Goat anti‐rabbit IgG HRP, MultiSciences).

For immunoprecipitation (IP) assays, cells were lysed in Co‐IP lysis buffer [50 mM Tris‐HCl, 5 mM EDTA, 150 mM NaCl, 0.5% (v/v) Nonidet‐P40, and 10% (v/v) glycerol (pH 7.4), supplemented with 1 mM PMSF, 1 mM Na3VO4, and 10 mM NaF]. The lysate was incubated with M2‐Flag beads (Sigma), anti‐His beads (MBL), anti‐HA beads (MBL) or anti‐Myc beads (Thermo Scientific) overnight at 4°C. The immunoprecipitates were washed at least three times in lysis buffer and then analysed by WB with the indicated antibodies (Flag, CST; His, MBL; HA, CST; Myc, Abmart).

### 2.18 Detection of in vivo neddylation modification

Cells were solubilized in Co‐IP lysis buffer containing the components listed above and 1% SDS (v/v) by rigorous scraping of the cells while on ice. For IP under denaturing conditions, the cell lysate was incubated at 100°C for 5 min. The lysate was then diluted 10 times with lysis buffer without SDS and IP with the indicated antibody mouse control IgG (Abclonal) and the anti‐Coro1a antibody (Santa Cruz) and the addition of protein A/G beads (Santa Cruz) or M2‐Flag beads (Sigma) alone overnight at 4°C. The immunocomplexes were then washed at least three times in lysis buffer, resolved by SDS‐PAGE, and analysed by WB with the indicated antibodies to Coro1a, NEDD8 (Abcam) and VeriBlot for IP Detection Reagent (HRP) (Abcam) or Flag (CST) and regular secondary antibodies.

### 2.19 In vitro neddylation assay

mCherry‐Coro1a‐Flag and mCherry‐TRIM4‐His were expressed in HEK293 cells and purified. For the neddylation assay, 0.5 μg of Flag‐Coro1a and 0.5 μg of His‐TRIM4 were incubated with 2 μg of His‐NEDD8, 10 ng of E1 (His‐APPBP1/UBA3) and 200 ng of E2 conjugating enzyme (His‐UBE2F or His‐UBE2M) in a total reaction volume of 20 μl and analysed with a Neddylation kit (Enzo Life Science). His‐UBE2F proteins were purchased from Proteintech. Flag‐Coro1a and His‐TRIM4 proteins were expressed and purified from HEK293 cells. Samples were incubated at 30°C for 1 h, and the reaction was terminated with the addition of SDS‐PAGE loading buffer without boiling before WB with the anti‐Flag (CST) antibodies.

### 2.20 Mass spectrometry

To analyse the neddylated proteins in EVs and the proteins interacting with Coro1a, anti‐NEDD8 IP EV proteins and proteins pulled down from cell lysates by anti‐Flag (Coro1a)‐coated beads were separated by SDS‐PAGE and stained with Coomassie brilliant blue R‐250 (Solarbio). Then, the gel was cut into 1 cm × 0.5 cm pieces. The protein content was analysed by mass spectrometry (MS) using the Q Exactive system (Thermo). To identify the lysine in Coro1a modified by a ubiquitination‐like process, NEDD8‐Coro1a was pulled down with M2 beads, and anti‐HA beads were then analysed by MS using the Q Exactive system. For EV‐WT and EV‐K233R analysis, a total of 100 μg of EV proteins was analysed by label‐free proteomics MS using the Q Exactive system.

### 2.21 Lentivirus and stable cell line construction

The production of lentivirus for MC38‐Coro1a or MC38‐Coro1a‐K233R overexpression was carried out as follows. HEK293 cells were seeded into one 10 cm dish. The following day, the cells were transfected with 5 μg of plvx‐mCherry‐Coro1a‐Flag or 5 μg of plvx‐mCherry‐Coro1a‐K233R‐Flag, 3.75 μg of psPAX2 (gag, pol) and 1.25 μg of pMD2.G using 20 μl of JetPEI. Viral supernatants were collected 48 h after transfection and filtered through 0.45 μm PVDF filters. For initial lentiviral transduction, MC38 cells were infected with the appropriate virus in six‐well plates in the presence of 10 μg ml^−1^ polybrene and centrifuged at 1500 × *g* for 2 h at 32°C. After 24 h, single mCherry^+^ cells were sorted into 96‐well plates with a Beckman MoFlo Astrios EQ (Beckman). The levels of endogenous and overexpressed proteins were then verified by WB.

For *Coro1a* knockout, HEK293 cells were seeded into 12‐well plates. The following day, the cells were cotransfected with plv5‐Cas9‐Blast (Merck) and U6‐gRNA: hPGK‐puro‐2A‐tBFP (Merck) including a specific gRNA sequence listed in Table S1. After 24 h, single BFP^+^ cells were sorted into 96‐well plates with a Beckman MoFlo Astrios EQ. The levels of endogenous and overexpressed proteins were then verified by WB.

### 2.22 Tumour models and treatments

Mice were subcutaneously injected with one million MC38, MC38‐*Coro1a^−/−^*, MC38‐Coro1a or MC38‐Coro1a‐K233R cells on day 0. The tumour‐bearing mice were randomized and injected with 20 μg of the indicated EVs via tail vein three times each week for 2 weeks beginning on day 1. Mouse cardiac blood was collected to detect the serum level of EVs by ELISA before an obvious difference in tumour size was observed on day 5. The mice were euthanized, and the draining lymph nodes (dLNs) were collected on day 20 for flow cytometry. The mice were considered to have “end‐stage” disease when the tumour reached 2 cm in at least one dimension. Tumour growth was monitored every 4 days by measuring tumour length and width. Tumour volume was calculated according to the following equation: length × width × 0.5 × width.

### 2.23 Flow cytometry of dLNs

The dLNs were extracted with sterilized surgical equipment and crushed with frosted surfaces in ice‐cold PBS. The cell mixtures were then filtered through 75 mm cell strainers into 15 ml conical tubes. The cells were washed, counted, and then seeded into 48‐well plates. Single‐cell suspensions (1 million cells) were first incubated with anti‐mouse Fc blocking antibodies (BioLegend) for 30 min at 4°C and then coincubated with Fixable Viability Dye (Invitrogen) to exclude dead cells followed by co‐incubated with antibodies against CD45 (BioLegend), CD3 (BioLegend), CD8 (Invitrogen), PD‐1 (BioLegend) and Tim‐3 (BioLegend) for 20 min at 4°C. For intracellular Gzm B (Invitrogen) staining, cells were stimulated for 4 h at 37°C in a medium containing PMA (Sigma, 50 ng ml^−1^), ionomycin (Sigma, 1 μg ml^−1^), and brefeldin A solution (Invitrogen), and then the cells were subjected to an intracellular staining protocol (Invitrogen). Intranuclear Ki‐67 (BioLegend) staining was carried out with fixation/permeabilization buffer solution (Invitrogen) according to the manufacturer's instructions. Flow cytometry was performed with an ACEA NovoCyte, and the data were analysed with NovoExpress software. The negative staining threshold was obtained with IgG isotype control (BioLegend).

### 2.24 Statistical analysis

Data are expressed as the mean ± s.d. Normal distribution of the data was analysed by Shapiro‐Wilk test. For normally distributed data, unpaired Student's *t*‐test was used for comparisons between two groups and ANOVA followed by Tukey multiple comparison test for comparisons among multiple groups. For data that were not normally distributed, Mann‐Whitney rank‐sum test was used for comparisons between two groups. Log‐rank test was used for survival rate analysis. All statistical analyses were performed using Graph Prism 8.0 software (GraphPad Software Inc., San Diego, CA, USA). Statistical significance was considered if a *P*‐value was < 0.05.

## 3 RESULTS

### 3.1 Neddylation inhibits EV secretion

**FIGURE 1:**
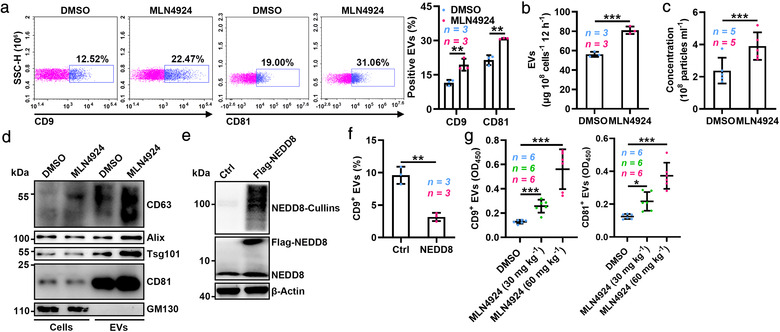
Neddylation inhibits EV secretion. (a) Flow cytometric analysis of EVs in the supernatants of HEK293 cells treated with DMSO or 100 nM MLN4924 for 12 h. Left, representative dot plots showing CD9 and CD81 staining of EVs captured with anti‑CD63‑coated beads after incubation with cell culture supernatants. Right, the ratio of CD9^+^ and CD81^+^ EVs. (b–d) EVs were purified from equal numbers of HEK293 cells treated with DMSO or 100 nM MLN4924 for 12 h. The BCA assay was used to determine the amount of EV protein (b). NTA to determine the EV concentration (c). WB analysis to detect the indicated EV markers (d). (e) WB analysis to detect the indicated proteins in HEK293 cells overexpressing NEDD8. (f) Flow cytometric ratio of CD9^+^ EVs in the supernatants of HEK293 cells overexpressing NEDD8. (g) ELISA analysis of CD9^+^ and CD81^+^ EVs in sera of mice intraperitoneally injected with MLN4924 at the indicated dose for 72 h. Representative results from three independent experiments are shown. *n*, sample number; *P < 0.05; **P < 0.01; ***P < 0.001 (unpaired two‐tailed Student's t‐test except for one‐way ANOVA followed by Tukey test in G; mean ± s.d.)

To determine the role of neddylation in EV production, we first treated HEK293, RAW264.7 and HeLa cells with MLN4924, a specific inhibitor of E1 NEDD8‐activating enzyme (NAE) that blocks neddylation modification (Soucy et al., 2009), and confirmed that 100 nM MLN4924 did not affect the proliferation or apoptosis of these cells within 12 h (Figure S1A, B). We also confirmed that 100 nM MLN4924 did not affect the CD9 and CD81 levels on individual EV from these cells (Figure S1C). Then, we measured EV secretion by these cells with a semiquantitative assay (Ostrowski et al., 2010) and found that MLN4924 significantly increased CD9^+^ and CD81^+^ EV secretion by these cells (Figure 1a, Figure S1D). Furthermore, the protein content and particle number of the EVs were also greatly increased in the MLN4924‐treated HEK293 cells (Figure 1b, c). Consistent with these findings, the signals for several markers enriched in exosomes (CD63, Alix, Tsg101 and CD81) were both notably enhanced in EVs from the same number of HEK293 cells with or without iodixanol gradient purification (Figure 1d and Figure S1E). Although MLN4924 promoted EV production, it did not alter the morphology or size distribution of the EVs (Figure S1F, G). Furthermore, silver staining revealed that MLN4924 did not globally affect the cell or EV protein content (Figure S1H).

To further confirm the effect of neddylation on EV secretion, we overexpressed NEDD8 in HEK293 cells and detected reduced EV secretion by these cells (Figure 1e, f). To exclude that free NEDD8 might regulate EV production, HEK293 cells were overexpressed with a NEDD8 mutant in which G76 was deficient (NEDD8‐ΔG76), resulting in disabling protein conjugation (Kandala et al., 2014). As expected, NEDD8‐ΔG76 overexpression did not reduce EV secretion (Figure S1I). In contrast, silencing of the catalytic subunit of the neddylation E1 enzyme UBA3 in HEK293 cells significantly increased EV production (Figure S1J, K). In addition, we found that MLN4924 dose‐dependently enhanced the quantity of serum CD9^+^ and CD81^+^ EVs in vivo (Figure 1g). Thus, our results demonstrate that neddylation suppresses EV secretion in vitro and in vivo.

### 3.2 Neddylation promotes the degradation of MVBs by lysosomes

**FIGURE 2:**
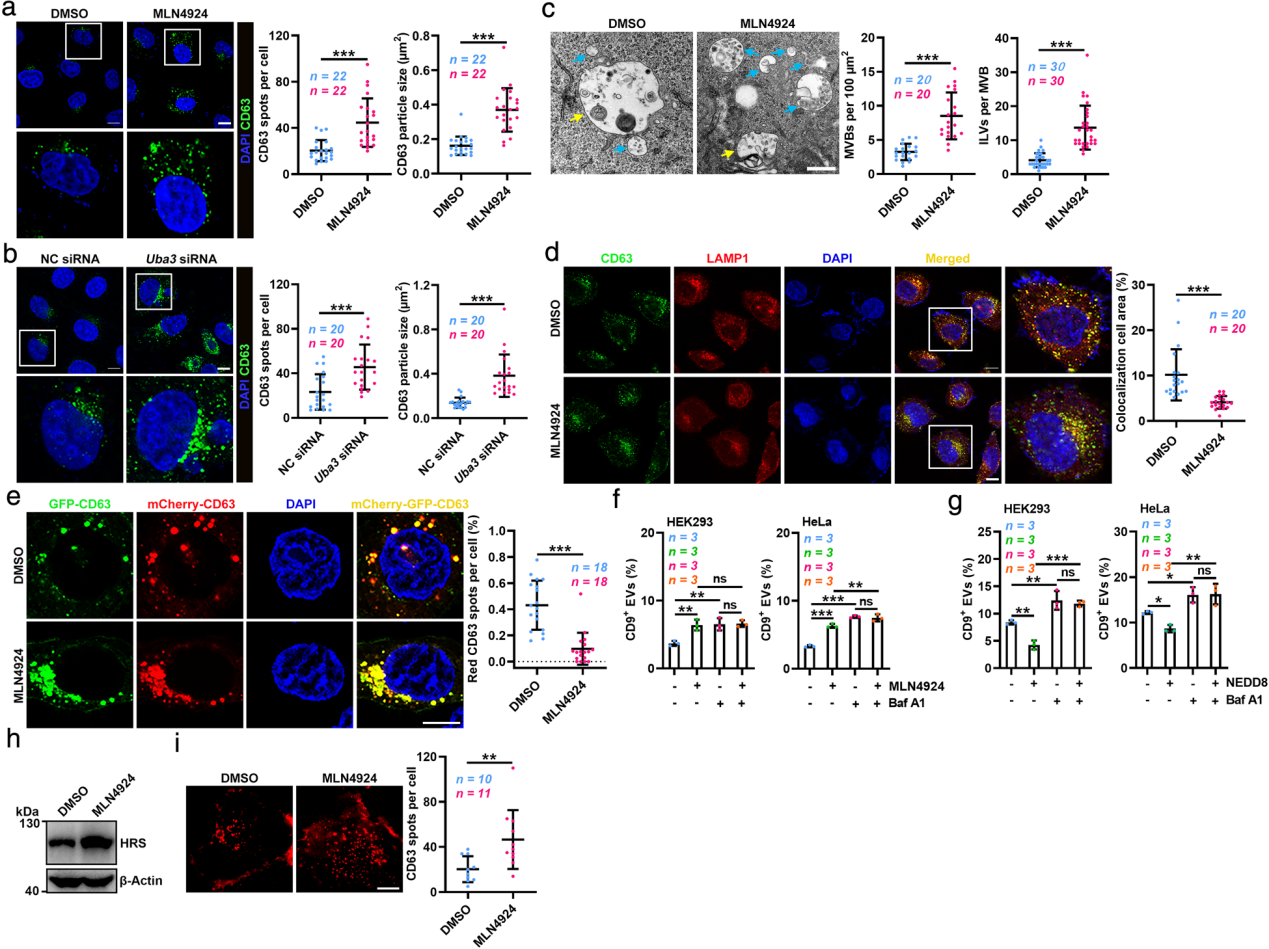
Neddylation promotes the lysosomal degradation of MVBs. (a, b) Left, confocal microscopy analysis of the MVB marker CD63 in HeLa cells treated with DMSO or 100 nM MLN4924 for 12 h (a) or HeLa cells transfected with NC or *Uba3* siRNA (B). Scale bar, 10 μm. Right panel, quantification of CD63^+^ spots and average particle size per cell. Each dot indicates the number of CD63^+^ spots and the average particle size per cell. (c) Left, EM images of MVBs (blue arrows) in HEK293 cells treated with DMSO or MLN4924 for 12 h. Scale bar, 500 nm. Yellow arrows indicate MVB and lysosome hybrids. Right, quantification of MVBs per cell per 100 μm^2^ and ILVs per MVB. Each dot indicates the number of MVBs per section and ILVs per MVB per cell. (d) Left, confocal microscopy analysis of CD63 and LAMP1 colocalization in HeLa cells treated with DMSO or 100 nM MLN4924 for 12 h. Scale bar, 10 μm. Right panel, each dot indicates the percentage of the colocalized area among the total LAMP1 area per cell. (e) Left, confocal microscopy analysis of mCherry‐GFP‐CD63 endosomes in HeLa cells treated with DMSO or 100 nM MLN4924 for 12 h. Scale bar, 10 μm. Right, the percentage of mCherry^+^GFP^−^ spots among the total mCherry^+^ spots per cell. Each dot indicates the proportion of red CD63 puncta per cell. (f, g) Flow cytometric ratio of CD9^+^ EVs in the supernatants of HEK293 and HeLa cells treated with 100 nM MLN4924 for 12 h (f) or overexpressing NEDD8 (g) in the presence of 20 nM Baf A1. (H) WB analysis of HRS in HEK293 cells treated with DMSO or 100 nM MLN4924 for 12 h. (i) Left, Representative TIRF microscopic images of the CD63^+^ MVB distribution in HeLa cells treated with DMSO or 100 nM MLN4924 for 12 h. Scale bar, 10 μm. Right, quantification of CD63^+^ spots in the subplasmalemmal region per cell. Each dot indicates the number of CD63^+^ spots per cell. Representative results from three independent experiments are shown. *n*, sample number; ns, not significant; *P < 0.05; **P < 0.01; ***P < 0.001 (unpaired two‐tailed Student's *t*‐test in A (left), B (left), C (left), I; unpaired Mann‐Whitney test in A (right), B (right), C (right), D, E; one‐way ANOVA followed by Tukey test in F, G; mean ± s.d.)

To explore how neddylation regulates EV secretion, we first investigated the effect of neddylation on MVBs by CD63, a well‐accepted MVB marker. We found that MLN4924 treatment significantly increased the number of CD63^+^ spots and enlarged the CD63^+^ spots of HeLa cells (Figure 2a). Similar results were observed with another MVB marker, hepatocyte growth factor‐regulated tyrosine kinase substrate (HRS) (Figure S2A). UBA3 silencing also increased the number of enlarged CD63^+^ spots in HeLa cells (Figure 2b). Moreover, the results of EM results revealed increased numbers of MVBs and ILVs in the MLN4924‐treated HEK293 cells (Figure 2c). This increase in MVBs might stem from accelerated biogenesis or reduced MVB degradation. EEA1 is a marker of EEs, and we found that MLN4924 did not affect the number of EEA1^+^ spots in HeLa cells (Figure S2B). Moreover, we transfected HeLa cells with the constitutively active Rab5^Q79L^ mutant, which results in the formation of large endosomes with a mixed morphology between EEs and MVBs, facilitating the observation of MVB biogenesis (Stenmark et al., 1994; Wegener et al., 2010). MLN4924 treatment did not affect the sorting of CD63 and lipid‐raft protein flotillin‐1 into Rab5^Q79L^ endosomes (Figure S2C, D). These results suggest that neddylation does not affect the MVB formation.

Then, we tested whether neddylation promotes the lysosomal degradation of MVBs. Upon a lysosomal marker LAMP1 staining, we found decreased colocalization of CD63 with lysosomes in MLN4924‐treated HeLa cells (Figure 2d). We further used the mCherry‐GFP‐CD63 construct to assess the fusion of MVBs and lysosomes. Due to the resistance of mCherry and sensitivity of GFP to low pH in lysosomes, GFP, but not mCherry, in MVBs was quenched after fusion with lysosomes. As expected, increased yellow puncta and decreased red puncta were observed in the MLN4924‐treated HeLa cells, indicating the decreased fusion of MVBs and lysosomes (Figure 2e). Furthermore, EM analysis showed that MLN4924 decreased the number of hybrids of MVBs and lysosomes that contained many electron‐dense vesicles (Figure S2E). These results indicate that MLN4924 prevents MVBs from lysosomal degradation.

Next, we tested the role of lysosomes in neddylation‐mediated regulation of EV production. Bafilomycin A1 (Baf A1) is a proton pump inhibitor that increases the pH of lysosomes and inhibits the fusion of MVBs and autophagsomes with lysosomes (Van Weert et al., 1995; vanDeurs et al., 1996). In the presence of Baf A1, neither MLN4924 treatment nor NEDD8 overexpression affected EV secretion by HEK293 and HeLa cells (Figure 2f, g). Similar results were obtained in the cells with Rab7a^T22N^ dominant‐negative overexpression (Figure S2F, G), which inhibited both endosome‐lysosome and autophagosome‐lysosome fusion (Bucci et al., 2000; Gutierrez et al., 2004). In addition, we found that MLN4924 treatment could still promote EV production in peritoneal macrophages from *Lc3b^−/−^* and *Beclin1* ^+/−^ mice (Figure S2H). These results indicate that neddylation‐mediated regulation of EV secretion depends on lysosomes but not autophagy.

MLN4924 enhanced EV production but still caused MVB accumulation, suggesting that the extent to which MVBs were increased due to reduced lysosomal degradation was higher than the extent to which the number of MVBs was decreased due to the fusion of MVBs and PM. If so, proteins in MVBs should accumulate after MLN4924 treatment. As expected, CD63 and HRS levels were substantially increased in the MLN4924‐treated HEK293 cells (Figure 1d and Figure 2h). Then, we used TIRF microscopy to evaluate the fusion of MVBs and PM. Along with the accumulation of CD63^+^ MVBs, more CD63^+^ MVBs were observed in the subplasmalemmal region of MLN4924‐treated HeLa cells (Figure 2i and Movies 1, 2). These results imply that accumulated MVBs cause increased exocytosis.

After docking to the PM, vesicle motion is restricted (Nofal et al., 2007). To exclude that MLN4924 promoted EV secretion by increasing the docking of MVBs and PM, the coefficient *D_xy_* of CD63^+^ MVBs, an index of mobility, was calculated along vesicle trajectories using a rolling analysis window (Ostrowski et al., 2010). Both *D_xy_* in HeLa cells treated with MLN4924 was not significantly different from that without MLN4924 treatment (Figure S2I), indicating that MLN4924 does not affect the docking of MVBs to the PM. Overall, these results demonstrate that neddylation decreases the number of MVBs by promoting MVB lysosomal degradation, resulting in reduced EV secretion.

### 3.3 Neddylation‐mediated inhibition of EV secretion is Coro1a dependent

**FIGURE 3:**
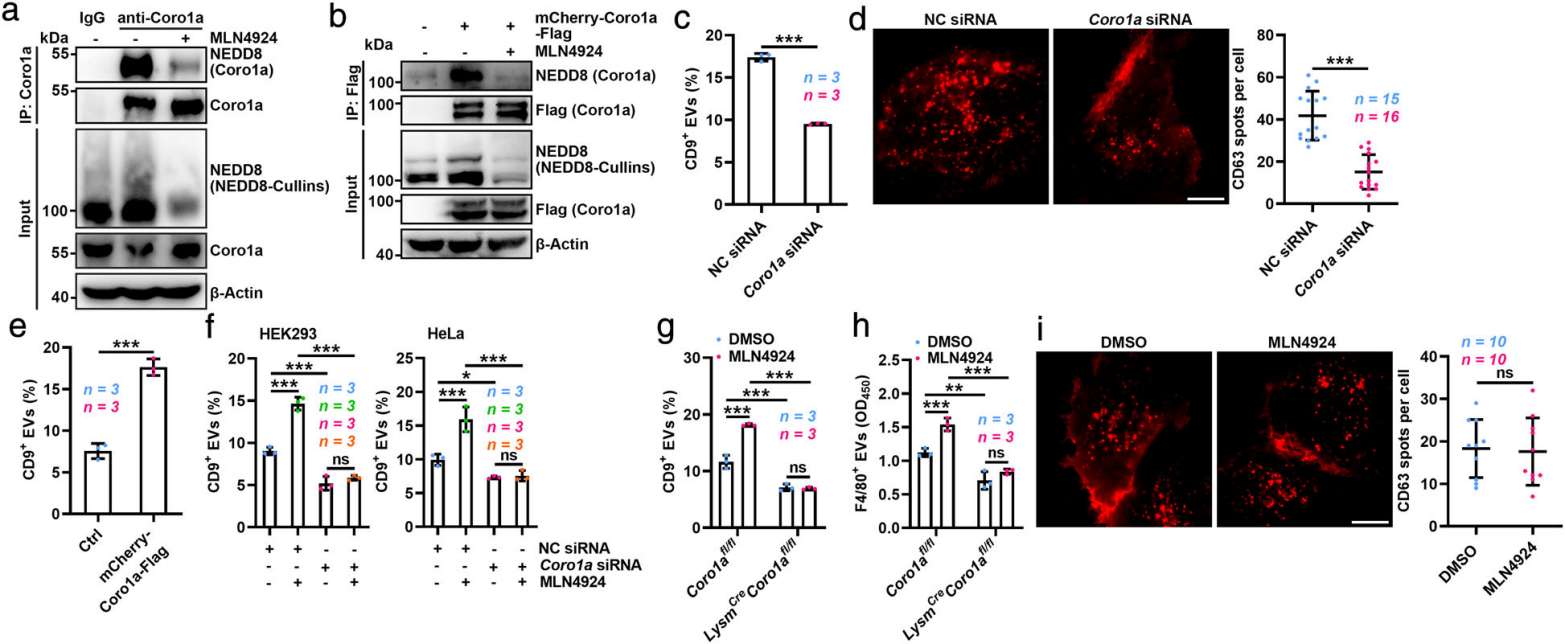
Neddylation‐mediated inhibition of EV secretion is Coro1a dependent. (a, b), WB analysis of NEDD8 and Coro1a in the lysates of HEK293 cells without (a) or with (b) mCherry‐Coro1a‐Flag overexpression followed by 100 nM MLN4924 treatment for 12 h after IP with anti‐Coro1a (a) or anti‐Flag (b). (c), Flow cytometric ratio of CD9^+^ EVs in the supernatants of HEK293 cells transfected with NC or *Coro1a* siRNA. (d), Left, representative TIRF microscopic images of the CD63^+^ endosome distribution in HeLa cells transfected with NC or *Coro1a* siRNA. Scale bar, 10 μm. Right panel, quantification of CD63^+^ vesicles in the subplasmalemmal region per cell. Each dot indicates the number of CD63^+^ vesicles per cell. (e) Flow cytometric ratio of CD9^+^ EVs in the supernatants of HEK293 cells with or without Coro1a overexpression. (f) Flow cytometric ratio of CD9^+^ EVs in the supernatants of HEK293 and HeLa cells transfected with NC or *Coro1a* siRNA and treated with or without 100 nM MLN4924 for 12 h. (g) Flow cytometric ratio of CD9^+^ EVs in the supernatants of BMDMs differentiated from the bone marrow cells of *Coro1a^fl/fl^* or *Lysm^Cre^Coro1a^fl/fl^* mice. (h) ELISA analysis of F4/80^+^ EVs in the sera of *Coro1a^fl/fl^* or *Lysm^Cre^Coro1a^fl/fl^* mice that intraperitoneally injected with 30 mg kg^−1^ MLN4924 for 72 h. (i) Left, representative TIRF microscopic images of the CD63^+^ endosome distribution in Coro1a‐silenced HeLa cells treated with DMSO or 100 nM MLN4924 for 12 h. Scale bar, 10 μm. Right panel, quantification of CD63^+^ vesicles in the subplasmalemmal region per cell. Each dot indicates the number of CD63^+^ vesicles per cell. Representative results from three independent experiments are shown. *n*, sample number; ns, not significant; *P < 0.05; **P < 0.01 and ***P < 0.001 (unpaired two‐tailed Student's *t*‐test in C, E and I; unpaired Mann‐Whitney test in D; one‐way ANOVA followed by Tukey test in F‐H; mean ± s.d.)

To dig out which neddylated protein(s) regulate EV secretion, we first tested the role of neddylated cullin3, a classic neddylation substrate, in EV secretion. DI‐591, a selective inhibitor of cullin3 neddylation (Zhou et al., 2017), obviously inhibited the neddylation of cullin3 but did not influence EV production (Figure S3A, B). Then, we analysed proteins of EVs from HEK293 cells with or without MLN4924 treatment by WB with anti‐NEDD8. Besides decreasing the signal of band (∼100 kD) representing NEDD8‐Cullins, MLN4924 treatment obviously reduced the signal of band between 40 and 55 kD (Figure S3C). Then, the neddylated proteins in these EVs were immunoprecipitated and analysed by MS. We found that the proteins between 40 and 55 kD included Coro1a (also termed TACO), which mediates the fusion of phagosomes and lysosomes (Ferrari et al., 1999) (Table S2). Therefore, we investigated whether Coro1a could undergo physiological neddylation indeed. Endogenous and exogeneous NEDD8‐Coro1a, which was sensitive to MLN4924 treatment, could be detected in HEK293 cells (Figure 3a, b). NEDD8‐Coro1a was also detected in HEK293 cells overexpressing NEDD8 but not NEDD8‐ΔG76 (Figure S3D). Moreover, overexpression of NEDP1, a NEDD8‐specific isopeptidase mediating substrate deneddylation (Zhao et al., 2014), or overexpression of two E1 subunits, UBA3 and NAE1, respectively, reduced and increased the NEDD8‐Coro1a in HEK293 cells (Figure S3E, F). Thus, these results indicate that Coro1a is a physiological substrate of neddylation.

Next, we determined the role of Coro1a in EV secretion, and first confirmed that the equal amounts of EVs from HEK293 cells with or without Coro1a silencing had similar levels of EV markers including CD63, Alix, Tsg101 and CD81 and similar size distribution (Figure S3G‐I). However, Coro1a silencing prominently inhibited EV production and reduced MVBs in the subplasmalemmal region (Figure 3c, d and Movie 3, 4). In contrast, overexpression of Coro1a promoted EV production (Figure 3e and Figure S3J). Neither MLN4924 treatment nor NEDD8 overexpression affected EV secretion in HEK293 or HeLa cells with Coro1a knockdown (Figure 3f and Figure S3K). Unexpectedly, in the presence of MLN4924, Coro1a silencing still greatly inhibited EV production (Figure 3f). Then, to further confirm the role of Coro1a in neddylation‐mediated EV generation, we constructed *Coro1a^fl/fl^* mice and crossed them with *Lysm^Cre^* mice to obtain *Lysm^Cre^Coro1a^fl/fl^* mice with Coro1a knockout in myelomonocytic cells including macrophages (Figure S3L, M). We found that MLN4924 treatment no longer increased EV secretion by Coro1a‐knockout macrophages in vitro or in vivo (Figure 3g, h). Similar to Coro1a silencing, MLN4924 could not abolish Coro1a deficiency‐induced decrease in EV secretion in vitro or in vivo (Figure 3g, h). However, upon Coro1a knockdown, we also found that MLN4924 no longer altered the MVB subplasmalemmal location (Figure 3i and Movie 5, 6). Altogether, these data indicate that Coro1a does not rely on neddylation to mediate EV biogenesis, but neddylation does regulate EV secretion in a Coro1a‐dependent manner.

### 3.4 UBE2F and TRIM4 are the E2 and E3 for Coro1a neddylation

**FIGURE 4:**
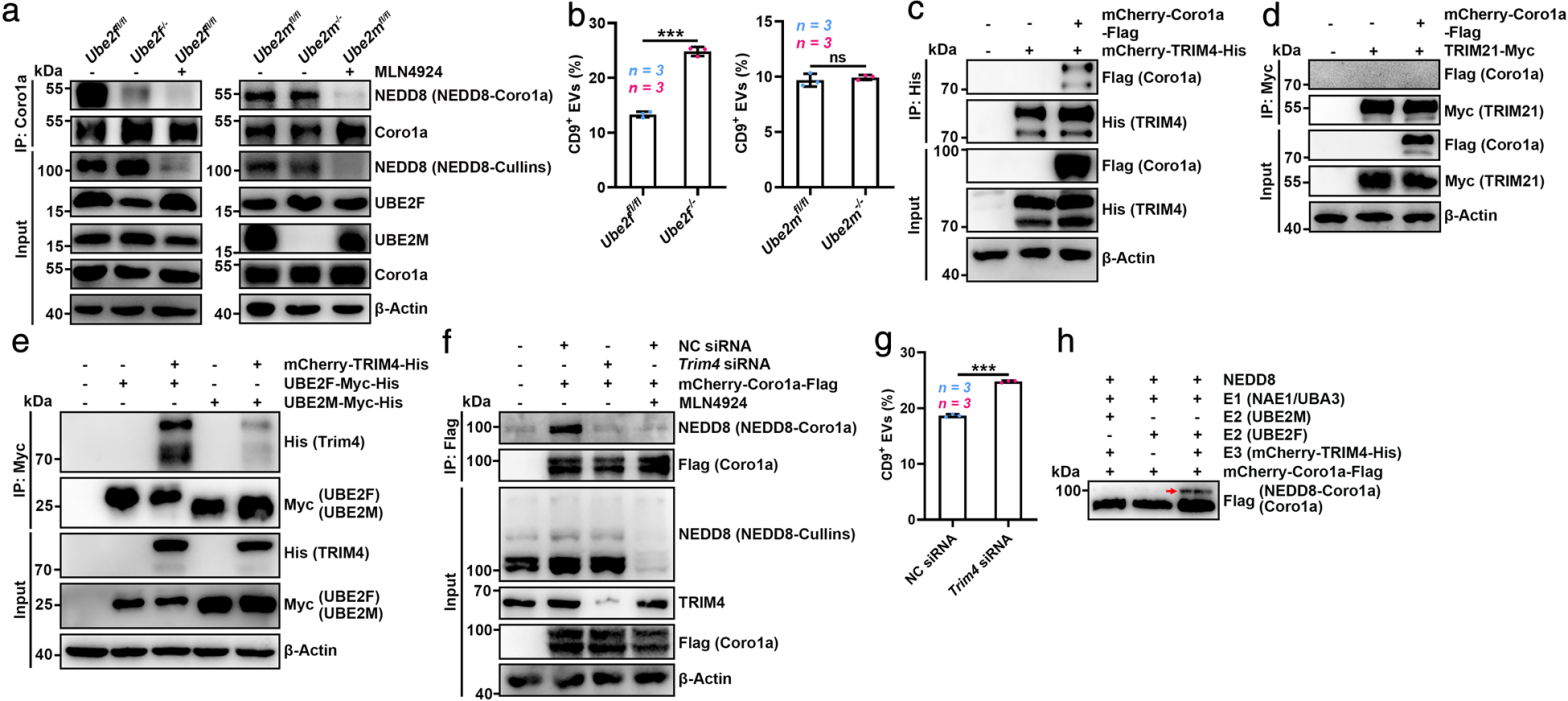
UBE2F and TRIM4 are the E2 and E3 for Coro1a neddylation. (a) WB analysis of NEDD8‐Coro1a and Coro1a in the lysates of *Ube2f^fl/fl^* BMDCs and *Ube2f^−/‐^* BMDMs or *Ube2m^fl/fl^* BMDCs and *Ube2m^−/‐^* BMDMs treated with or without 100 nM MLN4924 for 12 h after IP with anti‐Coro1a. (b) Flow cytometric ratio of CD9^+^ EVs in the supernatants of *Ube2f^fl/fl^* BMDCs and *Ube2f^−/‐^* BMDMs or *Ube2m^fl/fl^* BMDCs and *Ube2m^−/‐^* BMDMs. (c, d) WB analysis of Coro1a and TRIM4 (c) or Coro1a and TRIM21 (d) in the lysates of HEK293 cells overexpressing mCherry‐Coro1a‐Flag and mCherry‐TRIM4‐His (c) or mCherry‐Coro1a‐Flag and TRIM21‐Myc (d) after IP with anti‐His (c) or anti‐Myc (d). (e) WB analysis of TRIM4, UBE2M and UBE2F in the lysates of mCherry‐TRIM4‐His‐overexpressing HEK293 cells with UBE2F‐Myc‐His or UBE2M overexpression after IP with anti‐Myc. (f) WB analysis of NEDD8‐Coro1a and Coro1a in the lysates of HEK293 cells transfected with NC siRNA or *Trim4* siRNA and the mCherry‐Coro1a‐Flag expression vector with or without 100 nM MLN4924 treatment for 12 h after IP with anti‐Flag. (g) Flow cytometric ratio of CD9^+^ EVs in the supernatants of HEK293 cells transfected with NC or *Trim4* siRNA. (h) WB analysis of the in vitro neddylation of Coro1a in the presence of the purified NEDD8, NAE1 and UBA3 (E1); UBE2F or UBE2M (E2); mCherry‐TRIM4‐His (E3); and mCherry‐Coro1a‐Flag proteins. Red arrow indicated NEDD8‐Coro1a. Representative results from three independent experiments are shown. *n*, sample number; ns, not significant; ***P < 0.001 (unpaired two‐tailed Student's *t*‐test; mean ± s.d.)

UBE2F and UBE2M are two known E2 enzymes in the NEDD8 cascade (Huang et al., 2009). Therefore, we crossed the *Ube2f^fl/fl^* or *Ube2m^fl/fl^* mice (Wu et al., 2020) with *Lysm^Cre^* mice to obtain *Lysm^Cre^Ube2f^fl/fl^* (*Ube2f^−/−^*) or *Lysm^Cre^Ube2m^fl/fl^* (*Ube2m^−/−^*) mice in which UBE2F or UBE2M is deficient in myelomonocytic cells. Then, we investigated the roles of UBE2F and UBE2M in Coro1a neddylation by BMDMs differentiated from bone marrow cells of *Ube2f^fl/f^* and *Ube2f^−/‐^* or *Ube2m^fl/fl^* and *Ube2m^−/−^* mice (*Ube2f^fl/fl^* and *Ube2f^−/−^* BMDMs or *Ube2m^fl/fl^* and *Ube2m^−/−^* BMDMs) and confirmed that UBE2F was notably reduced in *Ube2f^−/−^* BMDMs while UBE2M was absent in *Ube2m^−/−^* BMDMs (Figure 4a). When compared to that in the corresponding E2 complete BMDMs, the level of NEDD8‐Coro1a was decreased in *Ube2f^−/−^* BMDMs but not *Ube2m^−/−^* BMDMs (Figure 4a). UBE2F but not UBE2M silencing also decreased exogenous NEDD8‐Coro1a in HEK293 cells (Figure S4A). In contrast, overexpression of UBE2F, but not UBE2M, obviously increased exogenous NEDD8‐Coro1a in HEK293 cells, which was greatly inhibited by MLN4924 (Figure S4B). Accordingly, *Ube2f^−/−^* BMDMs but not *Ube2m^−/−^* BMDMs secreted increased EVs (Figure 4b). In contrast, HEK293 cells overexpressing UBE2F but not UBE2M exhibited decreased EV production (Figure S4C). These results indicate that UBE2F is the E2 conjugating enzyme for Coro1a neddylation.

To identify the E3 ligase involved in Coro1a neddylation, we ectopically expressed Coro1a in HEK293 cells, followed by Coro1a pull down and MS analysis for Coro1a binding proteins. Three and ten specific peptides corresponding to the E3 ubiquitin ligases TRIM4 and TRIM21, respectively, were detected (Figure S4D and Table S3), as illustrated by representative MS spectra corresponding to ^173^KVMHLQDVEVK^183^ and ^311^LGDTQQSIPGNEER^324^, respectively (Figure S4E). Then, we tested whether TRIM4 and/or TRIM21 mediated Coro1a neddylation as an E3 ligase and found that TRIM4, but not TRIM21, interacted with Coro1a (Figure 4c, d). In addition, TRIM4 strongly interacted with UBE2F but hardly interacted with UBE2M (Figure 4e). TRIM4 silencing reduced NEDD8‐Coro1a, which was comparable to MLN4924 treatment (Figure 4f). In contrast, TRIM4 overexpression enhanced it, which was inhibited by MLN4924 (Figure S4F). As an E3 ubiquitin ligase, TRIM4 also increased the ubiquitination of Coro1a (Ub‐Coro1a) regardless of MLN4924 (Figure S4F). Consistently, TRIM4 silencing and overexpression promoted and inhibited EV production, respectively (Figure 4g, Figure S4G). Finally, in vitro neddylation experiment further confirmed that in the presence of NEDD8, an E1 enzyme (NAE1 and UBA3) and TRIM4 with UBE2F, but not with UBE2M, were sufficient to induce Coro1a neddylation (Figure 4h). Altogether, our results indicate that UBE2F serves as the E2 conjugating enzyme and TRIM4 serves as the E3 ligase for Coro1a neddylation.

### 3.5 Lysine 233 is the main neddylation site in Coro1a

**FIGURE 5:**
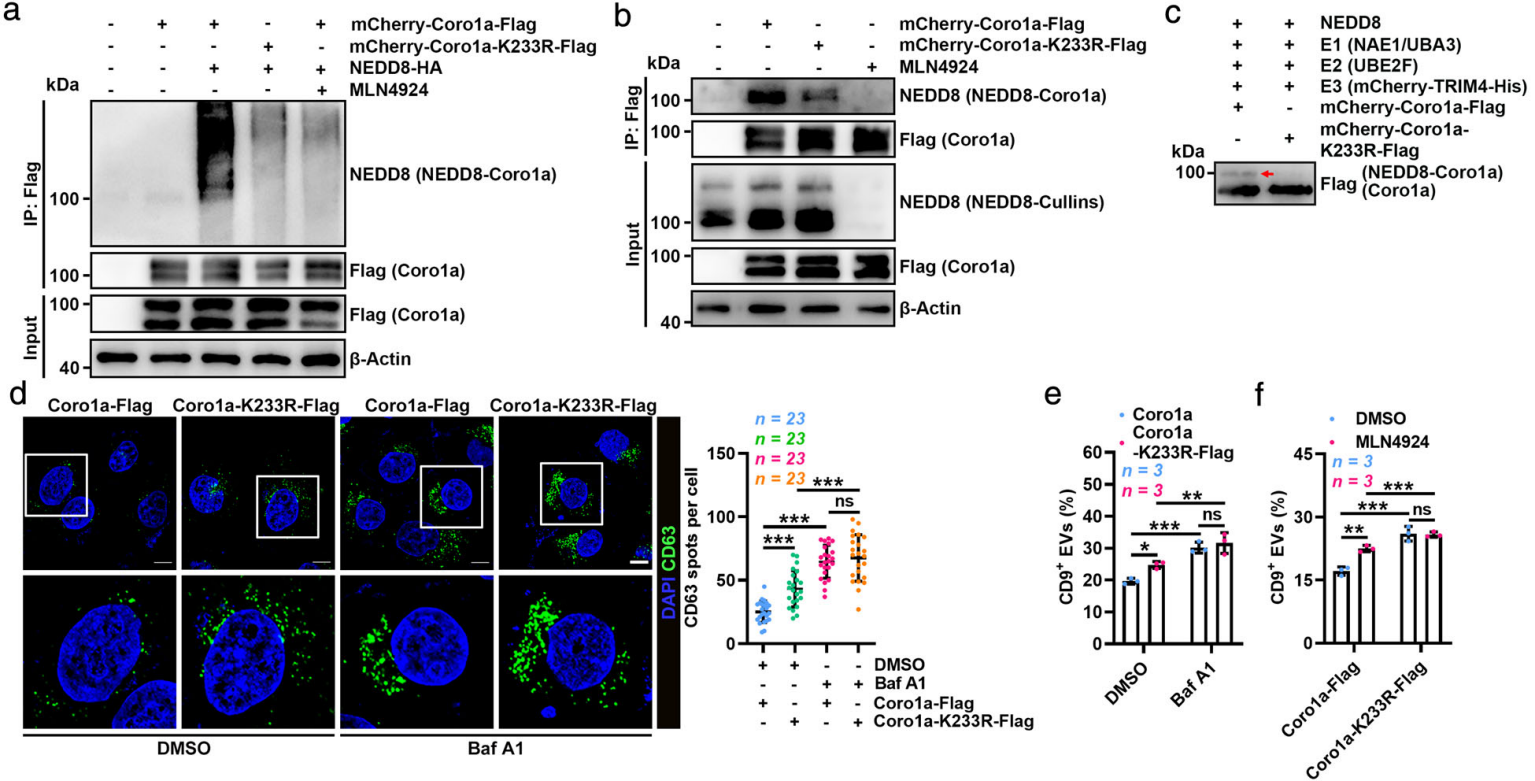
Lysine 233 is the main neddylation site in Coro1a. (a, b) WB analysis of NEDD8‐Coro1a and Coro1a in the lysates of HEK293 cells overexpressing mCherry‐Coro1a‐Flag or mCherry‐Coro1a‐K233R‐Flag and NEDD8‐HA (a), or mCherry‐Coro1a‐Flag or mCherry‐Coro1a‐K233R‐Flag (b) with or without 100 nM MLN4924 treatment after IP with anti‐Flag. (c) WB analysis of the in vitro neddylation of Coro1a in the presence of purified NEDD8, NAE1 and UBA3 (E1); UBE2F (E2); mCherry‐TRIM4‐His (E3); and mCherry‐Coro1a‐Flag or mCherry‐Coro1a‐K233R‐Flag proteins. Red arrow indicated NEDD8‐Coro1a. (d) Left, confocal microscopy analysis of the MVB marker CD63 in Coro1a‐Flag or Coro1a‐K233R‐Flag overexpressing HeLa cells treated with DMSO or 20 nM Baf A1 for 12 h. Scale bar, 10 μm. Right, quantification of CD63^+^ spots per cell. Each dot indicates the number of CD63^+^ spots per cell. (e, f) Flow cytometric ratio of CD9^+^ EVs in the supernatants of Coro1a‐Flag or Coro1a‐K233R‐Flag overexpressing HEK293 cells treated with DMSO, 20 nM Baf A1 (e) or 100 nM MLN4924 (f) for 12 h. Representative results from three independent experiments are shown. *n*, sample number; ns, not significant; *P < 0.05; **P < 0.01 and ***P < 0.001 (one‐way ANOVA followed by Tukey test; mean ± s.d.)

To determine the neddylation site(s) in Coro1a, we overexpressed Coro1a and NEDD8 in HEK293 cells and then pulled down Coro1a after proteins were dissociated, which was followed by another pulldown to enrich NEDD8‐Coro1a in immunoprecipitated Coro1a (Figure S5A). MS analysis of the enriched NEDD8‐Coro1a revealed a ubiquitination or ubiquitination‐like modification at K233 (Figure S5B and Table S4), a residue conserving in mammals (Figure S5C). Then, we mutated (K to R) K233 in Coro1a (Coro1a‐K233R) and found that this mutation obviously diminished Coro1a neddylation in HEK293 cells both with and without NEDD8 overexpression (Figure 5a, b). Furthermore, overexpression of UBE2F and TRIM4 only slightly increased Coro1a‐K233R neddylation while notably increased Coro1a neddylation (Figure S5D). We also obtained similar results in an in vitro neddylation experiment (Figure 5c). These results indicate that K233 is the neddylation site in Coro1a.

Next, we defined the biological relevance of K233 and found that Coro1a‐K233R overexpression increased accumulation of MVBs compared to that with Coro1a overexpression, which was abolished by Baf A1 (Figure 5d), suggesting a lysosome‐dependent mechanism. Accordingly, Coro1a‐K233R overexpression caused a lysosome‐dependent increase in EV secretion (Figure 5e). Moreover, MLN4924 promoted EV secretion in HEK293 cells overexpressing Coro1a but not Coro1a‐K233R (Figure 5f), further supporting that neddylation of Coro1a at K233 regulates EV production.

To evaluate the effect of NEDD8‐Coro1a on the EV protein composition, EVs from HEK293 cells overexpressing Coro1a or Coro1a‐K233R (EV‐WT or EV‐K233R, respectively) were analysed by MS (Table S5). The most abundant proteins in the EV‐WT and EV‐K233R were nearly identical (Figure S5E‐G). The levels of common markers enriched in exosomes differed by only a small amount between the EV‐WT and EV‐K233R (Figure S5H). In addition, the global levels of the 30 most often identified proteins in the ExoCarta exosome database and Coro1a were similar (Figure S5I). These results suggest that the K233R mutation in Coro1a had little effect on the overall protein composition of EVs.

### 3.6 NEDD8‐Coro1a mediates the recruitment of Rab7 to MVBs

**FIGURE 6:**
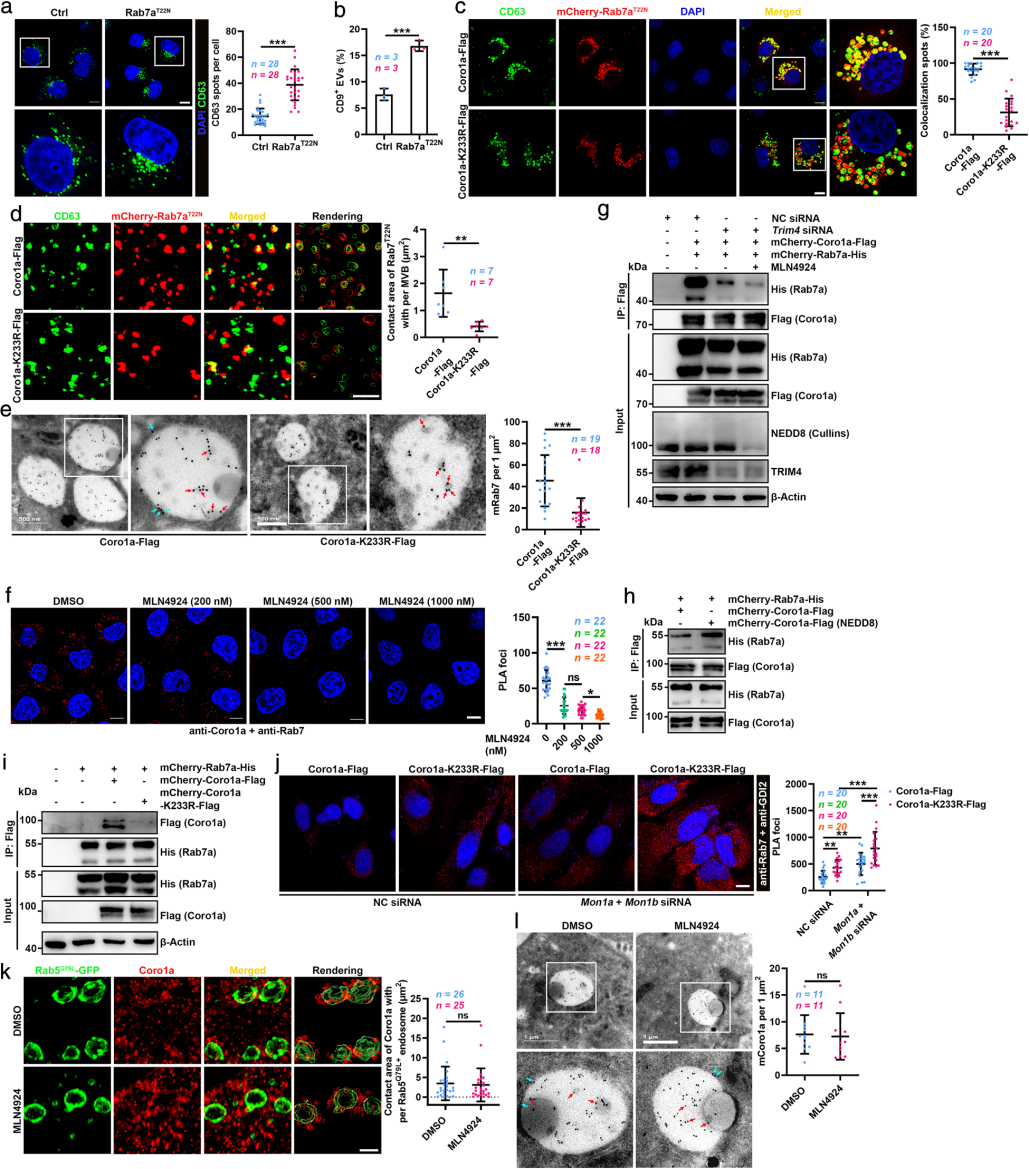
NEDD8‐Coro1a mediates the recruitment of Rab7 to MVBs. (a) Left, confocal microscopy analysis of CD63 in HeLa cells transfected with the Rab7a^T22N^ dominant‐negative mutant. Scale bar, 10 μm. Right, quantification of CD63^+^ spots per cell. Each dot indicates the number of CD63^+^ spots per cell. (b) Flow cytometric ratio of CD9^+^ EVs in the supernatants of HEK293 cells transfected with the Rab7a^T22N^. (c) Left, confocal microscopy analysis of CD63 and Rab7a^T22N^ colocalization in HeLa cells overexpressing mCherry‐Rab7a^T22N^ and Coro1a‐Flag or Coro1a‐K233R‐Flag. Scale bar, 10 μm. Right, quantification of spots showing colocalization per cell. Each dot indicates the percentage of spots per cell showing colocalization. (d) 3D‐SIM analysis of Rab7a^T22N^ on CD63^+^ MVBs of HeLa cells overexpressing mCherry‐Rab7a^T22N^ and Coro1a‐Flag or Coro1a‐K233R‐Flag and the corresponding reconstructed renderings by Imaris 9.5 (Rightmost). Yellow indicated contact surface. Scale bar, 3 μm. Right, quantification of the contact area of Rab7a^T22N^ with per MVBs. Each dot represents the mean contact area in each MVB from individual cell. (e) Left, electron micrographs of HEK293 cells overexpressing Coro1a‐Flag or Coro1a‐K233R‐Flag with anti‐Rab7 staining (conjugated with 18 nm gold). Representative mRab7 on MVBs (blue arrows) and ILVs (red arrows) are shown. Scale bars, 500 nm. Right panel, quantification of mRab7 gold particles on MVBs and ILVs per 1 μm^2^. Each dot indicates the number of mRab7 gold particles per section per cell. (f) Left, confocal microscopy analysis of PLA^+^ spots showing the interaction between Coro1a and Rab7 in HeLa cells treated with DMSO or MLN4924 at the indicated dose for 12 h. Scale bar, 10 μm. Right, quantification of PLA^+^ spots per cell. (g) WB analysis of Rab7a and Coro1a in the lysates of HEK293 cells transfected with vectors for mCherry‐Coro1a‐Flag and mCherry‐Rab7a‐His expression and NC or *Trim4* siRNA with or without 100 nM MLN4924 treatment for 12 h after IP with anti‐Flag. (h) WB analysis of the in vitro interaction between the purified mCherry‐Rab7a‐His protein and mCherry‐Coro1a‐Flag protein from HEK293 cells with mock vector transfection or the mCherry‐Coro1a‐Flag protein from HEK293 cells with NEDD8 vector transfection [mCherry‐Coro1a‐Flag (NEDD8)] after IP with anti‐Flag. (i) WB analysis of Coro1a and Rab7a in the lysates of HEK293 cells overexpressing mCherry‐Rab7a‐His and mCherry‐Coro1a‐Flag or mCherry‐Coro1a‐K233R‐Flag after IP with anti‐His. (j) Left, confocal microscopy analysis of PLA^+^ spots showing the interaction between Rab7a and GDI2 in HeLa cells overexpressing Coro1a‐Flag or Coro1a‐K233R‐Flag with or without Mon1a and Mon1b silencing. Scale bar, 10 μm. Right, quantification of PLA^+^ spots per cell. (k) The images of Coro1a in Rab5^Q79L^ endosomes of HeLa cells treated with DMSO or 100 nM MLN4924 for 12 h analysed by 3D‐SIM and the corresponding reconstructed renderings by Imaris 9.5 (Rightmost). Yellow indicated contact surface. Scale bar, 3 μm. Right, quantification of the contact area of Coro1a with per Rab5^Q79L^ endosome. Each dot represents the total contact area of Coro1a^+^ spots per Rab5^Q79L^ endosome. (l) Left, electron micrographs of HEK293 cells treated with DMSO or 100 nM MLN4924 for 12 h followed by anti‐Coro1a staining (conjugated with 18 nm gold). Representative mCoro1a on MVBs (blue arrows) and ILVs (red arrows) are shown. Scale bars, 1 μm. Right, quantification of Coro1a gold particles per MVB per 1 μm^2^. Each dot indicates the number of Coro1a gold particles per section per cell. Representative results from three independent experiments are shown. *n*, sample number; ns, not significant; *P < 0.05; *P* < 0.01; and ***P < 0.001 (unpaired Mann‐Whitney test in A, C‐E, K, L; unpaired two‐tailed Student's *t*‐test in B; one‐way ANOVA followed by Tukey test in F, J; mean ± s.d.)

Given that neddylation promoted MVB degradation by lysosomes, we assumed that NEDD8‐Coro1a increased the fusion of MVBs and lysosomes. Rab7 acts in LE‐to‐lysosome transport (Meresse et al., 1995). Rab7a^T22N^ overexpression increased the accumulation of MVBs and EV secretion (Figure 6a, b). The opposite results were obtained upon overexpression of the active Rab7a^Q67L^ mutant (Figure S6A, B). These results indicate that Rab7 negatively regulates EV secretion. Then, we investigated whether NEDD8‐Coro1a controls EV secretion by affecting Rab7 recruitment to MVBs. To ease the detection of CD63^+^ spots, we overexpressed Rab7a^T22N^ in HeLa cells and then assessed the MVB localization of Rab7. Compared with Coro1a overexpression, Rab7a had reduced MVB localization in HeLa cells with Coro1a‐K233R overexpression (Figure 6c). To exclude that the T22N mutation affects the MVB localization of Rab7, we treated HeLa cells with Baf A1 to enlarge the MVBs, and the detected results were similar to those obtained by Rab7a^T22N^ overexpression (Figure S6C). 3D‐SIM revealed that Coro1a‐K233R overexpression decreased membrane‐associated Rab7a (mRab7a) on MVBs (Figure 6d). In addition, immuno‐EM results confirmed that Coro1a‐K233R overexpression significantly reduced mRab7 on MVBs and ILVs (Figure 6e). These results indicate that NEDD8‐Coro1a contributes to Rab7 recruitment onto MVBs.

Mon1 including Mon1a and Mon1b is the only protein found to mediate Rab7 recruitment to endosomes in mammalian cells (Kinchen & Ravichandran, 2010; Poteryaev et al., 2010). Mon1a and Mon1b silencing indeed decreased the MVB localization of Rab7a^T22N^ in HeLa cells (Figure S6D, E). Consistently, increased EV secretion by Mon1a and Mon1b‐silenced HEK293 cells was observed (Figure S6F). However, MLN4924 still decreased the MVB localization of Rab7a^T22N^ and increased EV secretion by these cells (Figure S6G, H), suggesting Mon1 is dispensable in this process. In addition, MLN4924 and Mon1a and Mon1b silencing showed synergy effect on promoting EV secretion (Figure S6H). Therefore, we further explored how NEDD8‐Coro1a recruits Rab7 to MVBs. PLA revealed the direct interaction between Coro1a and Rab7, which was dose‐dependently inhibited by MLN4924 (Figure 6f). Moreover, Coro1a could IP Rab7a, which was markedly inhibited by TRIM4 silencing and further inhibited by MLN4924 combinatorial treatment (Figure 6g). Similar results were obtained when Coro1a was IP by Rab7a (Figure S6I). More directly, the Coro1a and Rab7a proteins pulled down each other in vitro, which was promoted by the neddylation of Coro1a (Figure 6h and Figure S6J). However, Rab7a hardly interacted with Coro1a‐K233R (Figure 6i). These results suggest that NEDD8‐Coro1a recruits Rab7 by direct binding. After its recruitment to endosomes, Rab7 is activated by dissociating from Rab guanine nucleotide dissociation inhibitor (GDI). Compared to Coro1a overexpression, Coro1a‐K233R overexpression increased the association of GDI2 and Rab7a independent of Mon1 (Figure 6j). Moreover, Mon1a and Mon1b silencing further enhanced this effect (Figure 6j). Binding of Rab7 to its effector RILP indicates Rab7 activation (Sun et al., 2007). The association of RILP with Rab7 was also reduced in HeLa cells overexpressing Coro1a‐K233R (Figure S6K). In addition, Coro1a had higher avidity to active Rab7a (Figure S6L). Altogether, these results demonstrate that NEDD8‐Coro1a directly binds Rab7 and induces its activation independent of Mon1.

Since Coro1a recruits Rab7 to MVBs by direct interaction, Coro1a should localize on the MVB membrane. Both membrane‐associated and cytoplasmic Coro1a and Rab7 were observed upon detection by WB, and the ratio of mCoro1a to soluble Coro1a was not affected by MLN4924, while the ratio of mRab7 to soluble Rab7 was affected by MLN4924 (Figure S6M, N). 3D‐SIM detected Coro1a on Rab5^Q79L^ endosomes which did not affect by MLN4924 either (Figure 6k). Similarly, immuno‐EM showed that Coro1a could locate on MVBs and ILVs independent of MLN4924 (Figure 6l). Furthermore, mCoro1a was detected on CD63^+^ EVs, and CD63^+^ EVs could be captured by anti‐Coro1a (Figure S6O, P). Overall, neddylated MVB‐localized mCoro1a recruits Rab7 to MVBs by directly binding Rab7.

### 3.7 Coro1a is an ideal target for the regulation of EV secretion

Finally, we wanted to test whether Coro1a is an ideal target for the regulation of EV biogenesis. First, we confirmed that the enrichment of Coro1a in the iodixanol gradient fraction was similar to that of marks enriched in exosomes (Alix, CD63 and CD81) (Figure S7A). In addition, Annexin A1, a specific marker of microvesicles (Jeppesen et al., 2019), was undetectable in Coro1a^+^ EVs (Figure S7B). These results show that Coro1a can act as a novel marker of EVs and may hold potential in the discrimination of exosomes and microvesicles.

**FIGURE 7:**
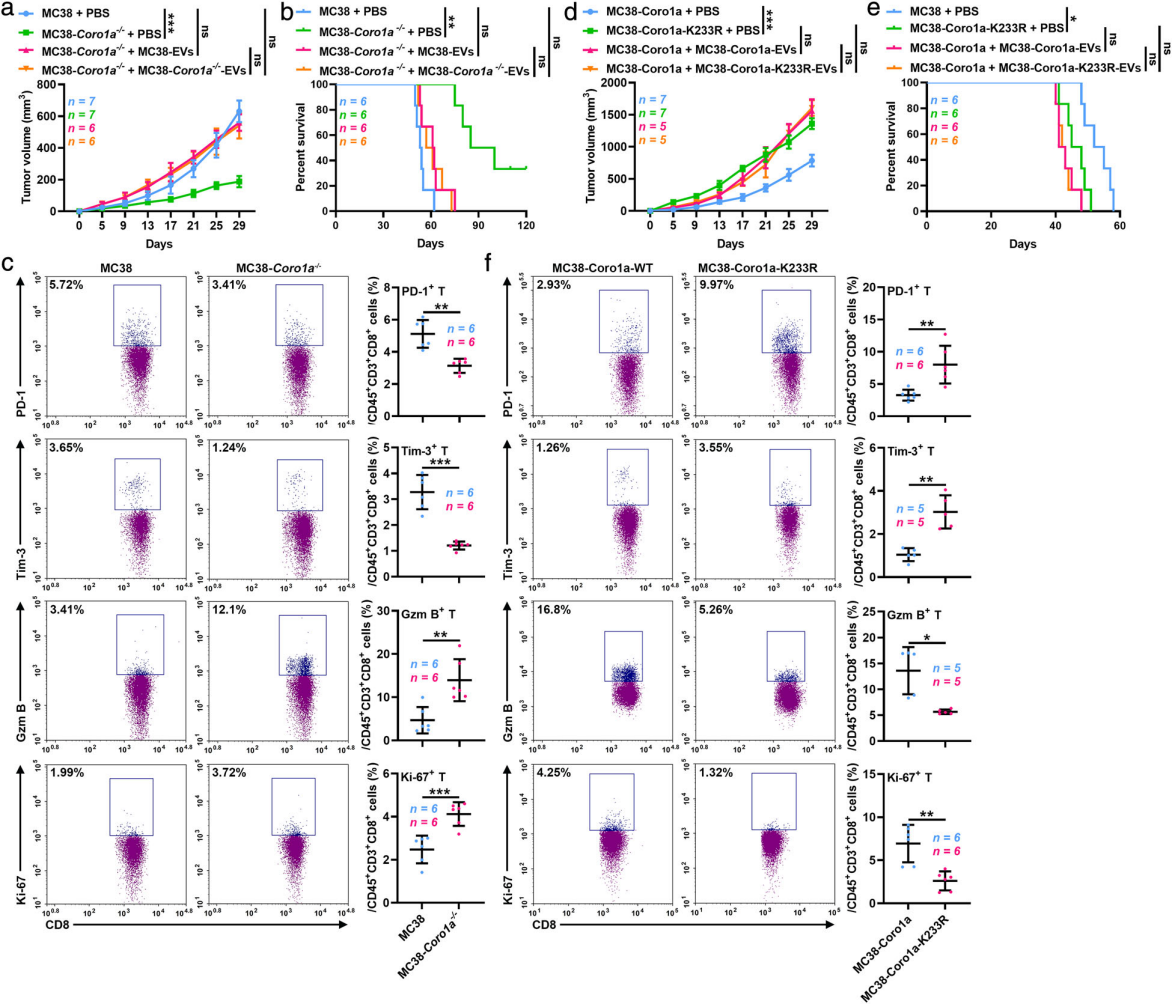
Coro1a is an ideal target for the regulation of EV secretion. (a, b) Tumour sizes (a) and survival curves (b) of mice that received 1 × 10^6^ MC38 or MC38‐*Coro1a^−/−^* cells by subcutaneous injection with or without the intravenous injection of 20 μg of the indicated EVs 3 times per week for 2 weeks. (c) Flow cytometric quantification of the percentages of cells expressing PD‐1, Tim‐3, Gzm B and Ki‐67 among the CD45^+^CD3^+^CD8^+^ T cell populations in dLNs from MC38 or MC38‐*Coro1a^−/−^* tumour‐bearing mice on day 20. (d, e) Tumour sizes (d) and survival curves (e) of mice that received 1 × 10^6^ MC38‐Coro1a or MC38‐Coro1a‐K233R cells by subcutaneous injection with or without the intravenous injection of 20 μg the indicated EVs 3 times per week for 2 weeks. (f) Flow cytometric quantification of the percentages of cells expressing PD‐1, Tim‐3, Gzm B and Ki‐67 among the CD45^+^CD3^+^CD8^+^ T cell populations in dLNs from MC38‐Coro1a or MC38‐Coro1a‐K233R tumour‐bearing mice. Representative results from three independent experiments are shown. *n*, sample number; ns, not significant; *P < 0.05, **P < 0.01 and ***P < 0.001 (one‐way ANOVA followed by Tukey test in A, D; log‐rank test in B, E; unpaired two‐tailed Student's *t*‐test in C, F; mean ± s.d.)

A decrease in EV production by MC38 colon cancer cells upon Rab27a knockout led to the release of systemic immunosuppression (Poggio et al., 2019). We then investigated tumour growth when Coro1a was used as a target to regulate EV secretion. We constructed murine MC38 colon cancer cells with Coro1a knockout (MC38‐*Coro1a^−/−^*) (Figure S7C), Coro1a overexpression (MC38‐Coro1a) and Coro1a‐K233R overexpression (MC38‐Coro1a‐K233R). EV production was significantly decreased in MC38‐*Coro1a^−/−^* cells and increased in MC38‐Coro1a‐K233R cells (Figure S7D). In addition, EV secretion notably increased in MC38 cells with Coro1a or Coro1a‐K233R overexpression when compared with MC38 cells (Figure S7D). Compared with that in MC38 tumour‐bearing mice, tumour growth in *Coro1a^−/−^* tumour‐bearing mice was greatly retarded, accompanied by a marked increase in survival (Figure 7a, b). In *Coro1a^−/−^* tumour‐bearing mice, the proportion of CD8^+^ T cells in the dLNs was significantly increased (Figure S7E). In addition, decreased exhaustion markers and increased activation and proliferation markers were detected in CD8^+^ T cells (Figure 7c). Supplementation with the same quantity of EVs from MC38 cells (MC38‐EVs) and from MC38‐*Coro1a^−/‐^* cells (MC38‐*Coro1a^−/−^*‐EVs) equally rescued the growth inferiority of *Coro1a^−/−^* tumour and shortened the survival (Figure 7a, b), suggesting that Coro1a knockout did not alter EV function. Conversely, MC38‐Coro1a‐K233R tumour‐bearing mice exhibited faster tumour growth and shorter survival than MC38‐Coro1a tumour‐bearing mice (Figure 7d, e). Furthermore, the function and proliferation CD8^+^ T cells were inhibited, and the proportion of total CD8^+^ T cells in the dLNs was decreased in MC38‐Coro1a‐K233R tumour‐bearing mice (Figure 7f and Figure S7F). Before an obvious difference in tumour size was observed, the quantity of serum EVs was significantly reduced in MC38‐*Coro1a^−/−^* tumour‐bearing mice but enhanced in MC38‐Coro1a‐K233R tumour‐bearing mice (Figure S7G, H). Similarly, supplementation with the same quantity of EVs from MC38‐Coro1a (MC38‐Coro1a‐EVs) and from MC38‐Coro1a‐K233R cells (MC38‐Coro1a‐K233R‐EVs) abolished the growth priority of MC38‐Coro1a‐K233R tumour and equally prolonged the survival (Figure 7d, e). Collectively, these data suggest that tumour development can be affected by the Coro1a‐mediated regulation of EV secretion.

## 4 DISCUSSION

The regulation of EV biogenesis is a promising strategy for the treatment of various diseases due to the important effects of EVs on the progression of related diseases (Jiang et al., 2016; Kalluri & LeBleu, 2020; Zhang et al., 2019). Here, we demonstrate that protein neddylation plays an important role in the biogenesis of EVs. We have revealed that Coro1a undergone neddylation and that the localization of NEDD8‐Coro1a on MVBs promotes the recruitment of Rab7 onto MVBs by directly binding Rab7. Then, more MVBs are targeted to lysosomes for degradation, leading to the inhibition of EV secretion. Consistent with these findings, MLN4924, a specific inhibitor of neddylation, was found to markedly increase EV secretion in vitro and in vivo. When measurement of the MLN4924 effect on EVs secretion in vivo, MLN4924 was intraperitoneally administered which would cause extensive inhibition of EV production from multiple cells (including various parenchymal cells, epithelial cells and immune cells etc.). Given that the EVs in peripheral blood have multiple cellular origins, a significant decrease in total amount of serum EVs could be detected after MLN4924 treatment. In addition to the application in basic research, several phase I/II clinical trials of MLN4924 for anticancer applications are underway (Zhou et al., 2018), suggesting the biosafety of MLN4924. Therefore, MLN4924 probably is an ideal promoter of EV secretion. However, MLN4924 should be carefully selected when used in tumour study due to its potential antitumour effects. In addition, considering the tumour promoting function of EVs, the anticancer effect of MLN4924 may be blunted by the increased EV secretion, an unexpected side effect.

Under conditions in which excess NEDD8 is produced, such as the overexpression of exogenous NEDD8, the ubiquitin E1 enzyme can activate NEDD8, leading to the conjugation of NEDD8 to ubiquitylation substrates through the ubiquitin pathway, but this does not occur under physiological conditions. Therefore, a genuine neddylation substrate should adhere to three criteria: (1) covalent attachment of NEDD8 through its carboxy‐terminal G to a K residue on the target protein, (2) detectable neddylation under homeostatic conditions and with endogenous levels of NEDD8 and substrate expression, and (3) sensitivity to treatment with MLN4924 (Enchev et al., 2015). We found the conjugation of Coro1a with NEDD8 but not NEDD8‐ΔG76, the neddylation of Coro1a under physiological conditions, which was greatly suppressed by MLN4924 and UBE2F and TRIM4 are the E2 conjugating enzyme and E3 ligase, respectively, involved in Coro1a neddylation. The regulatory effect of Coro1a neddylation on EV biogenesis was revealed as well. Collectively, these findings demonstrate that Coro1a is a genuine substrate of neddylation.

Rab7 regulates trafficking from EEs to LEs/MVBs and trafficking between LEs/MVBs and lysosomes (Bucci et al., 2000; Rink et al., 2005). Therefore, inactivation of Rab7 should either reduce EV secretion by decreasing MVB biogenesis or promote EV secretion by decreasing MVB degradation. Nevertheless, our results showed that the inactivation of Rab7 promoted EV secretion, while the activation of Rab7 inhibited EV secretion, which is consistent with a previous publication in which Rab7 was concluded to be dispensable for trafficking from EEs to LEs/MVBs but required for the fusion of LEs/MVBs and lysosomes (Vanlandingham & Ceresa, 2009). This is further supported by results of increase in EV production from cells treated with Baf A1, which can also prevent the fusion of MVBs and lysosomes similar to the inactivated Rab7. In addition, ILVs were still observed when the conversion of EEs to LEs/MVBs was prevented by expression of the constitutively active Rab5^Q79L^ mutant, suggesting that Rab7 is not required for the formation of ILVs. Hence, we consider that MVBs are not just the intersection with the LEs but are the mixtures of EEs and LEs. The substitution of Rab7 for Rab5 on MVBs is likely to provide a lysosome‐targeting signal for MVBs but not affect the biogenesis of MVBs.

Although Mon1 has been reported to recruit Rab7 to endosomes, Mon1 cannot directly bind Rab7 but interacts with the core of HOPS tethering complex of Rab7 (Kinchen & Ravichandran, 2010; Poteryaev et al., 2010). Alternatively, the interaction between Rab7 and Mon1 could only be detected after Mon1 formed a complex with its binding partner, Ccz1 (Kinchen & Ravichandran, 2010). Our results demonstrate that silencing of Mon1a and Mon1b inhibited the colocalization of Rab7 and MVBs which supports the function of Mon1 on endosome location of Rab7. Consistent with the critical role of Rab7 in MVB and lysosome fusion which leads to MVB degradation and subsequent EV secretion, silencing of Mon1a and Mon1b notably promoted EV secretion. However, silencing of Mon1a and Mon1b did not abolish MLN4924 to promote EV secretion, suggesting a Mon1‐independent regulation of EV production by MLN4924. In addition, MLN4924 and Mon1 had synergy effect on promoting EV secretion. Thus, these functions of MLN4924 and Mon1 are not redundant. Given that MLN4924 increased EV secretion depending on NEDD8‐Coro1a and NEDD8‐Coro1a can directly bound Rab7. Therefore, NEDD8‐Coro1a represents a novel mechanism responsible for the localization of Rab7 at LEs and MVBs, which is critical for the transport of LEs and MVBs to lysosomes and thus determines MVB fate and EV biogenesis. To recruit Rab7, membrane location is necessary. Although we have revealed that Coro1a is membrane‐associated, Coro1a has not trasmembrane domain. Thence, how Coro1a is located on membrane is unknown. Probably, it binds to certain unidentified membrane protein(s) which is interesting and deserves further study.

The mammal guanine nucleotide exchange factor (GEF) of Rab7 is still unraveled so far. As the GEF of Rab7 in yeast, Vps39 cannot function as the GEF for mammal Rab7 (Peralta et al., 2010). Although Mon1‐Ccz1 complex is the GEF of yeast Rab7 (Nordmann et al., 2010), orthologues of mammalian Ccz1 is still unknown. According to our results, inhibition of Coro1a neddylation reduced Rab7 activation dispensable for Mon1. Moreover, inhibition of Coro1a neddylation and silencing of Mon1a and Mon1b also exhibited synergy effect on Rab7 inactivation. This shows that NEDD8‐Coro1a and Mon1 represent non‐redundant pathways of Rab7 activation. Therefore, NEDD8‐Coro1a probably is a promising candidate for the mammal Rab7 GEF.

Tumour EVs promote tumour progression by inhibiting antitumour immunity. Our data showed that MC38 cells expressing Coro1a with deficient neddylation were more malignant and exhibited weaker antitumour immunity through their secretion of more EVs, which implies that the induction of NEDD8‐Coro1a is a promising strategy to improve antitumour immunity. In addition, we found that the knockout of Coro1a greatly inhibited tumour progression and enhanced antitumour immunity by reducing EV secretion. Based on our results, Coro1a silencing and knockout still suppressed EV secretion in the presence of MLN4924. Therefore, Coro1a regulates EV biogenesis in both neddylation‐dependent and neddylation‐independent manners. This further emphasizes the profound effects of Coro1a on EV biogenesis, although we did not determine how Coro1a regulates EV biogenesis independent of neddylation in this study. In addition to its role as a target in tumours, Coro1a probably holds high potential as a target in other diseases in which EVs are involved.

## AUTHOR CONTRIBUTIONS

ZJ.C. designed and supervised the research. JL.W., ZM.Y., J.Z. and Y.S. co‐supervised the research. XF.F., ZJ.L., DY.Y., XH.K., XL.L., YY.S., JL.W. and YC.Z. performed the experiments. X.L. and SF.X. performed the MS analysis. XF.F. and ZJ.C. analysed the data and wrote the paper.

## CONFLICTS OF INTEREST

The authors report no conflicts of interest.

## Supporting information

Supporting Information

Supporting Information

Supporting Information

Supporting Information

Supporting Information

Supporting Information

Supporting Information

Supporting Information

Supporting Information

Supporting Information

Supporting Information

Supporting Information

## Data Availability

The data for the study are available from the corresponding author upon request.
