# Supplementary material for: Neddylation of Coro1a determines the fate of multivesicular bodies and biogenesis of extracellular vesicles
Source: J Extracell Vesicles. 2021 Oct 8;10(12):e12153. doi: 10.1002/jev2.12153 (PMC8500273; doi:10.1002/jev2.12153)
Supplement: Supplementary file 1 — Supporting Information [file JEV2-10-e12153-s001.pdf]

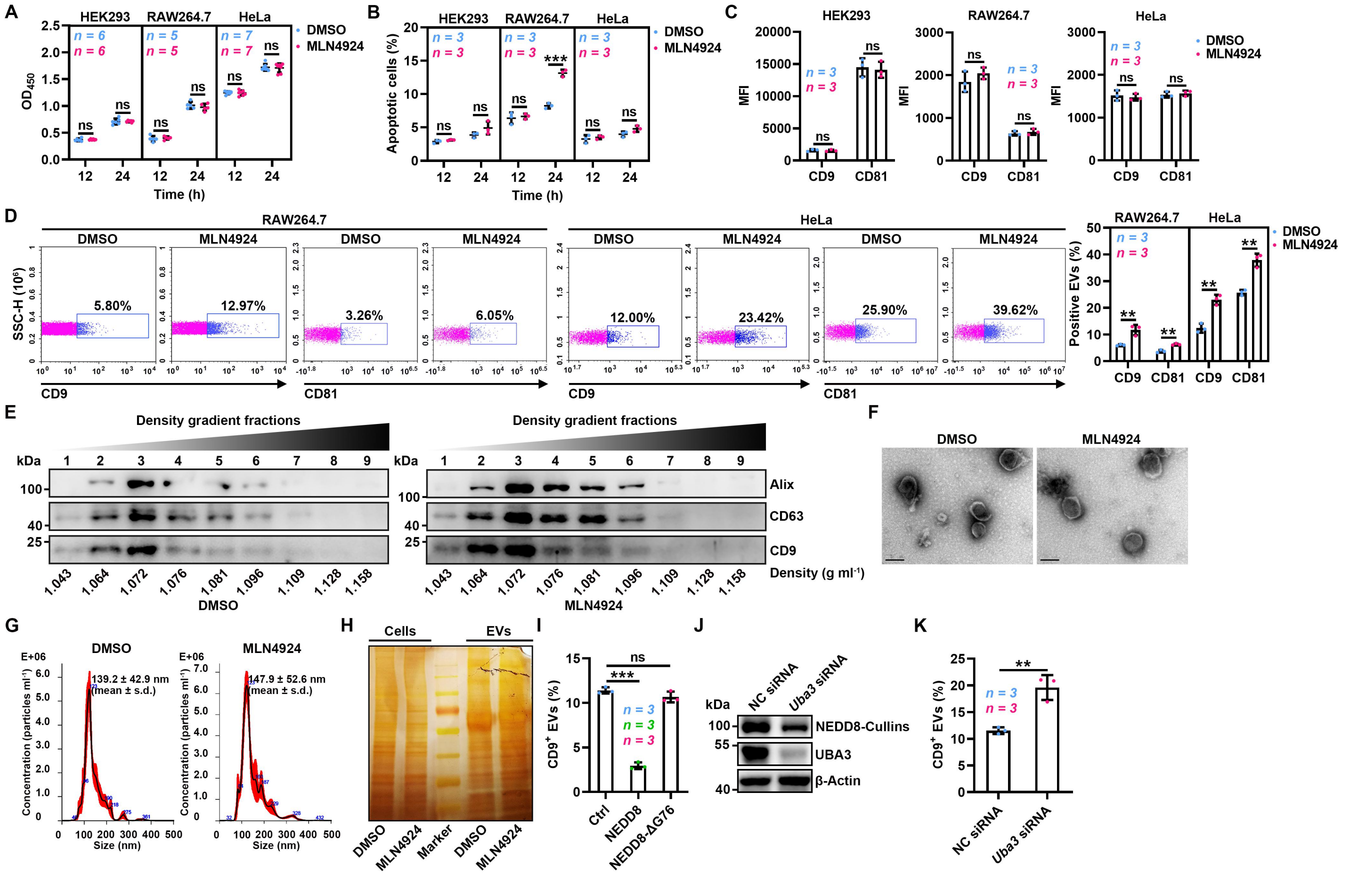

Supplementary Figure 1: Neddylation inhibits EV secretion

(*A*, *B*) CCK-8 assay to detect proliferation (*A*) and flow cytometric analysis of apoptosis (Annexin V^{+}) (*B*) in HEK293, RAW264.7 and HeLa cells treated with DMSO or 100 nM MLN4924 for 12 or 24 h. (*C*) Flow cytometric detection of CD9 and CD81 levels on latex beads coated with equal amount of EVs from HEK293, RAW264.7 or HeLa cells treated with DMSO or 100 nM MLN4924 for 12 h. MFI, mean fluorescence intensity. (*D*) Representative dot plots and the ratio of CD9^{+} and CD81^{+} EVs after incubation with supernatants from RAW264.7 or HeLa cells treated with DMSO or 100 nM MLN4924 for 12 h. (*E*) WB analysis of the indicated EV markers in different iodixanol gradient fractions obtained from HEK293 cell supernatants. (*F*, *G*) EM (*F*) and NTA (*G*) analysis of EVs from HEK293 cells treated with DMSO or 100 nM MLN4924 for 12 h. (*H*) Silver staining of equal amounts of HEK293 cells treated with DMSO or 100 nM MLN4924 for 12 h or EVs from the corresponding cells. (*I*) The ratio of CD9^{+} EVs in the supernatants of HEK293 cells overexpressing NEDD8 or NEDD8-ΔG76. (*J*, *K*) WB

analysis (*J*) and the ratio of $CD9^+$ EVs in the supernatants (*K*) of HEK293 cells transfected with negative control (NC) or *Uba3* siRNA. Representative results from three independent experiments are shown. n, sample number; ns, not significant; $^{**}P < 0.01$ and $^{***}P < 0.001$ (unpaired two-tailed Student's t-test except for one-way ANOVA followed by Tukey test in *I*; mean ± s.d.).

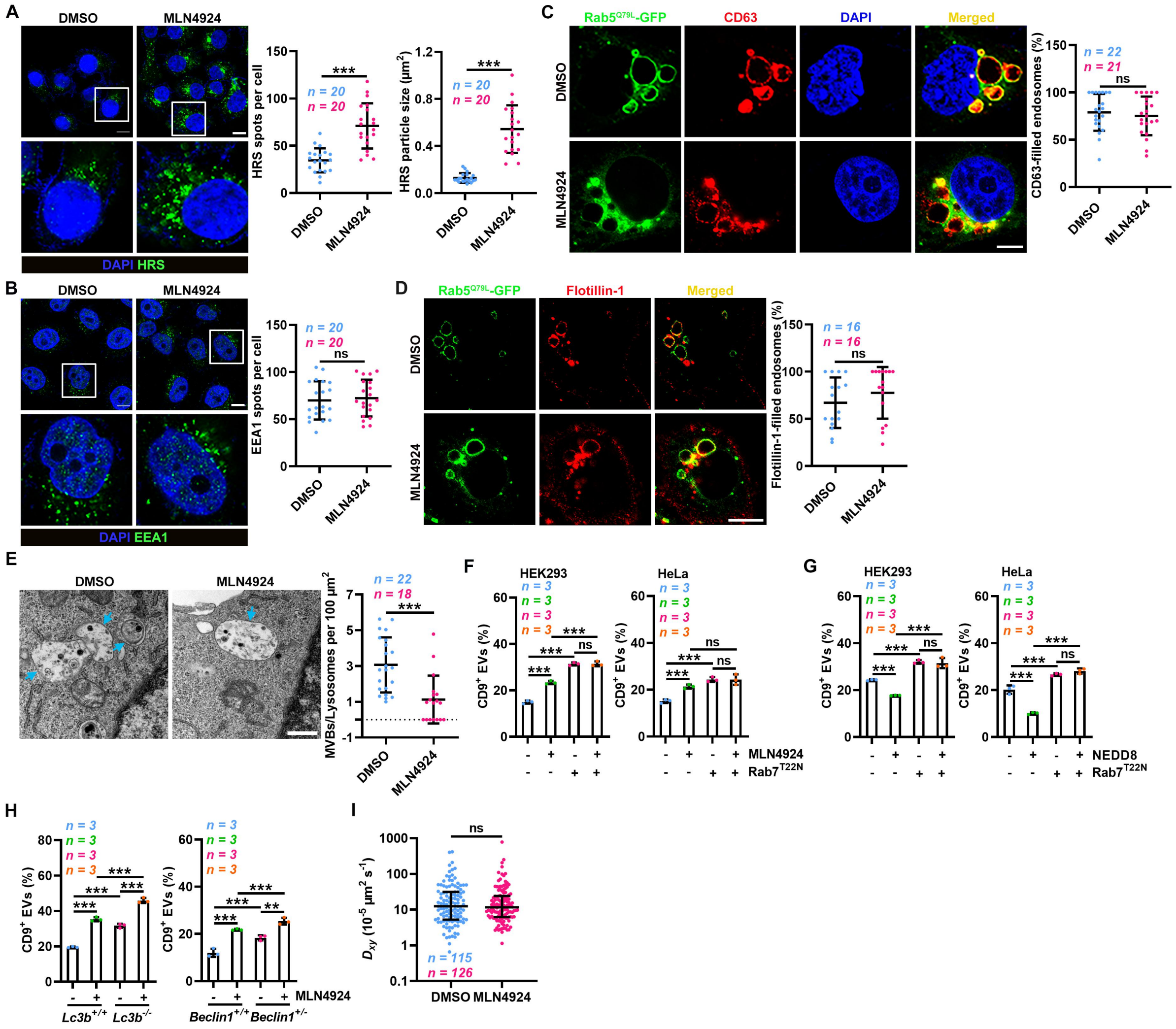

Supplementary Figure 2: Neddylation promotes the lysosomal degradation of MVBs

(*A*, *B*) Left, confocal microscopy analysis of the MVB marker HRS (*A*) and the EE marker EEA1 (*B*) in HeLa cells treated with DMSO or 100 nM MLN4924 for 12 h. Scale bar, 10 µm. Right panel, quantification of HRS^+ spots and average particle size or $EEA1^+$ spots per cell. Each dot indicates the number of spots and average particle size per cell. (*C*, *D*) Left, confocal microscopy analysis of CD63 (*C*) or Flotillin-1 (*D*) sorting into $Rab5^{Q79L}$-GFP endosomes in HeLa cells transfected with $Rab5^{Q79L}$-GFP and then treated with DMSO or MLN4924 for 12 h. Scale bar, 10 µm. Right panel, each dot indicates the percentage of CD63- or Flotillin-1-filled endosomes per cell. (*E*) Left, EM images of MVB and lysosome hybrids (blue arrows) in HEK293 cells treated with DMSO or MLN4924 for 12 h. Scale bar, 500 nm. Right graph, quantification of MVB and lysosome hybrid structures per section. Each dot

indicates the number of hybrids per cell per 100 μm^2. (*F*, *G*) Flow cytometric ratio of CD9$^+$ EVs in the supernatants of HEK293 and HeLa cells transfected with the Rab7a^{T22N} dominant-negative mutant and treated with 100 nM MLN4924 for 12 h (*F*) or those overexpressing NEDD8 (*G*). (*H*) Flow cytometric ratio of CD9$^+$ EVs in the supernatants of *Lc3b*$^{-/-}$ and *Beclin1*$^{+/-}$ BMDMs treated with or without 100 nM MLN4924 for 12 h. (*I*) Effect of MLN4924 treatment on CD63$^+$ MVB motion. Mean diffusion coefficient (*Dxy*) values for the individual trajectories of at least 100 endosomes in DMSO- or MLN4924-treated HeLa cells are shown. Representative results from three independent experiments are shown. *n*, sample number; ns, not significant; $^{**}P < 0.01$; $^{***}P < 0.001$ (unpaired two-tailed Student's *t*-test in *A* (left), *B*; unpaired Mann-Whitney test in *A* (right), *C*-*E*, *I*; one-way ANOVA followed by Tukey test in *F*-*H*; mean ± s.d. except for median ± interquartile ranges in *I*).

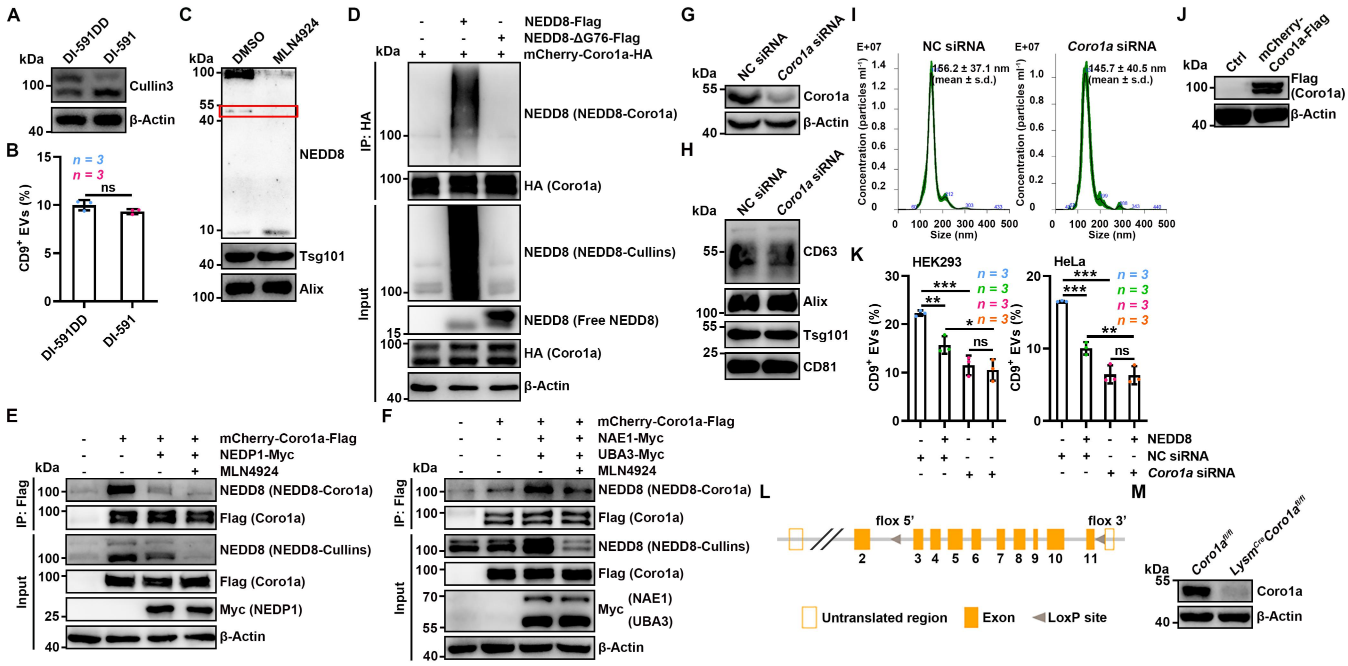

Supplementary Figure 3: Neddylation-mediated inhibition of EV secretion is Coro1a dependent

(*A*, *B*) WB analysis (*A*) and the flow cytometric ratio of CD9$^+$ EVs in the supernatants (*B*) of HEK293 cells treated with 10 μM DI-591DD (control) or DI-591 for 12 h. (*C*) WB analysis of NEDD8 proteins in EVs purified from the supernatants of HEK293 cells treated with DMSO or 100 nM MLN4924 for 12 h. (*D*-*F*) WB analysis of NEDD8-Coro1a and Coro1a in the lysates of HEK293 cells transfected with vectors for NEDD8-Flag or NEDD8-ΔG76-Flag and mCherry-Coro1a-HA (*D*) or mCherry-Coro1a-Flag and NEDP1-Myc expression that were then treated with or without 100 nM MLN4924 for 12 h (*E*) or mCherry-Coro1a-Flag, NAE1-Myc and UBA3-Myc expression that were then treated with or without 100 nM MLN4924 for 12 h (*F*) after IP with anti-HA (*D*) and anti-Flag (*E*, *F*). (*G*-*I*) WB analysis of Coro1a in HEK293 cells transfected with NC or *Coro1a* siRNA (*G*) or CD63, Alix, Tsg101 and CD81 in the equal amounts of EVs from these cells (*H*), or NTA analysis of the equal amounts of EVs from these cells (*I*). (*J*) WB analysis of HEK293 cells transfected with or without mCherry-Coro1a-Flag overexpression. (*K*) Flow cytometric ratio of CD9$^+$ EVs in the supernatants of NEDD8-overexpressing HEK293 and HeLa cells transfected with NC or *Coro1a* siRNA. (*L*) Knockout strategy in *Coro1a*$^{fl/fl}$

mice. LoxP sites were inserted such that they flanked exons 3 and 11. (*M*) WB analysis of Coro1a in BMDMs differentiated from the bone marrow cells of $Coro1a^{fl/fl}$ or $Lysm^{Cre}Coro1a^{fl/fl}$ mice. Representative results from three independent experiments are shown. *n*, sample number; ns, not significant; *P < 0.05, **P < 0.01 and ***P < 0.001 (unpaired two-tailed Student's *t*-test except for one-way ANOVA followed by Tukey test in *K*; mean ± s.d.).

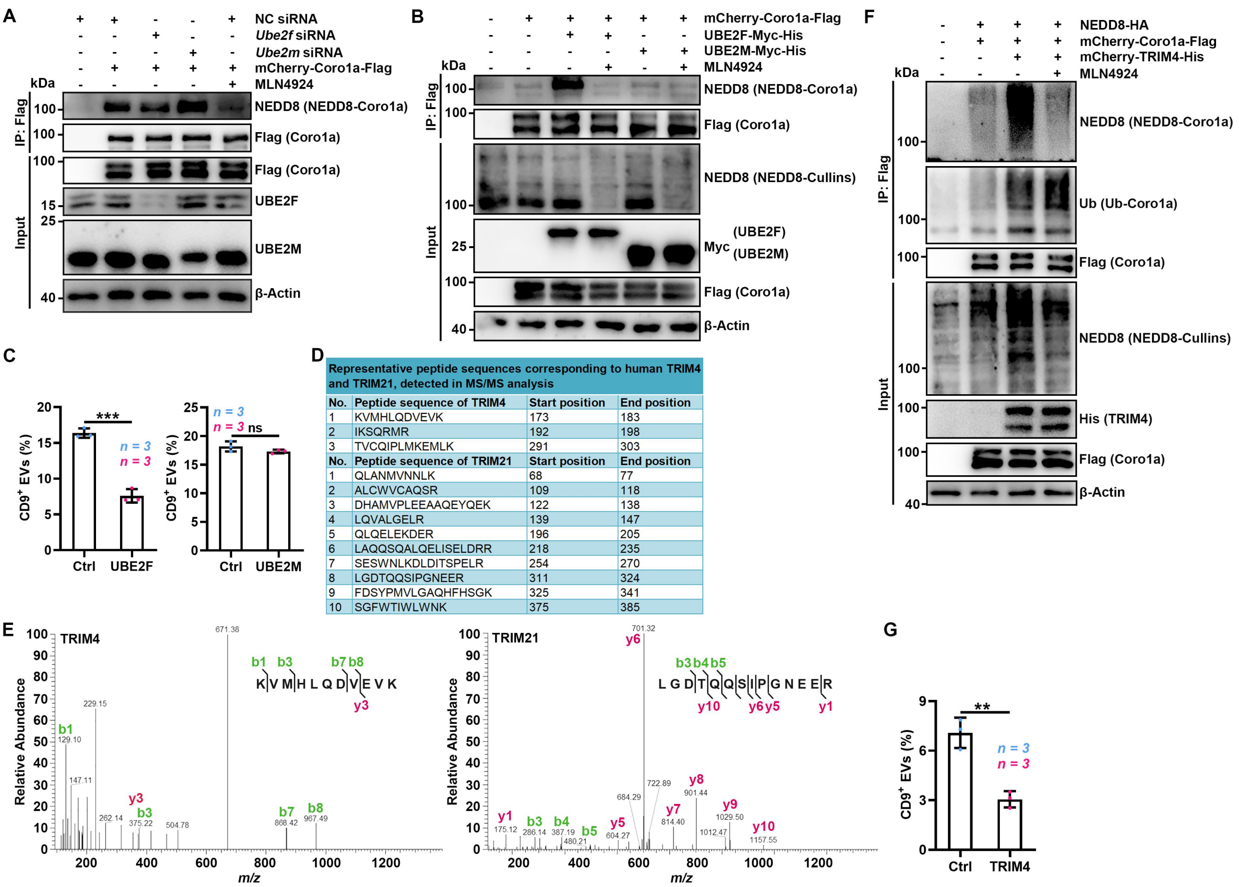

Representative peptide sequences corresponding to human TRIM4 and TRIM21, detected in MS/MS analysis			
No.	Peptide sequence of TRIM4	Start position	End position
1	KVMHLQDVEVK	173	183
2	IKSQRMR	192	198
3	TVCQIPLMKEMLK	291	303
No.	Peptide sequence of TRIM21	Start position	End position
1	QLANMVNNLK	68	77
2	ALCWVCAQSR	109	118
3	DHAMVPLEEAAQEYQEK	122	138
4	LQVALGELR	139	147
5	QLQELEKDER	196	205
6	LAQQSQALQELISELDRR	218	235
7	SESWNLKDLDITSPELR	254	270
8	LGDTQQSIPGNEER	311	324
9	FDSYPMVLGAQHFHSGK	325	341
10	SGFWTIWLWNK	375	385

Supplementary Figure 4: UBE2F and TRIM4 are the E2 and E3 for Coro1a neddylation

(*A*) WB analysis of NEDD8-Coro1a and Coro1a in the lysates of HEK293 cells transfected with NC, *Ube2f* or *Ube2m* siRNA and the mCherry-Coro1a-Flag expression vector with or without 100 nM MLN4924 treatment for 12 h after IP with anti-Flag. (*B*) WB analysis of NEDD8-Coro1a and Coro1a in the lysates of HEK293 cells transfected with vectors for mCherry-Coro1a-Flag and UBE2F-Myc-His or UBE2M-Myc-His expression with or without 100 nM MLN4924 treatment for 12 h after IP with anti-Flag. (*C*) Flow cytometric ratio of CD9^{+} EVs in the supernatants of HEK293 cells overexpressing UBE2F or UBE2M. (*D*) Peptide sequences corresponding to the E3 ubiquitin ligases TRIM4 and TRIM21 in the lysates of HEK293 cells overexpressing mCherry-Coro1a-Flag after IP with anti-Flag, followed by MS analysis. (*E*) Representative MS/MS spectra of TRIM4 and TRIM21 with the indicated b- and y-ion series. (*F*) WB analysis of NEDD8-Coro1a, Ub-Coro1a and Coro1a in the lysates of

HEK293 cells transfected with vectors for NEDD8-HA, mCherry-Coro1a-Flag and mCherry-TRIM4-His expression with or without 100 nM MLN4924 treatment for 12 h after IP with anti-Flag. (*G*) Flow cytometric ratio of $CD9^+$ EVs in the supernatants of HEK293 cells overexpressing TRIM4. Representative results from three independent experiments are shown. *n*, sample number; ns, not significant; $^{**}P < 0.01$ and $^{***}P < 0.001$ (unpaired two-tailed Student's *t*-test; mean ± s.d.).

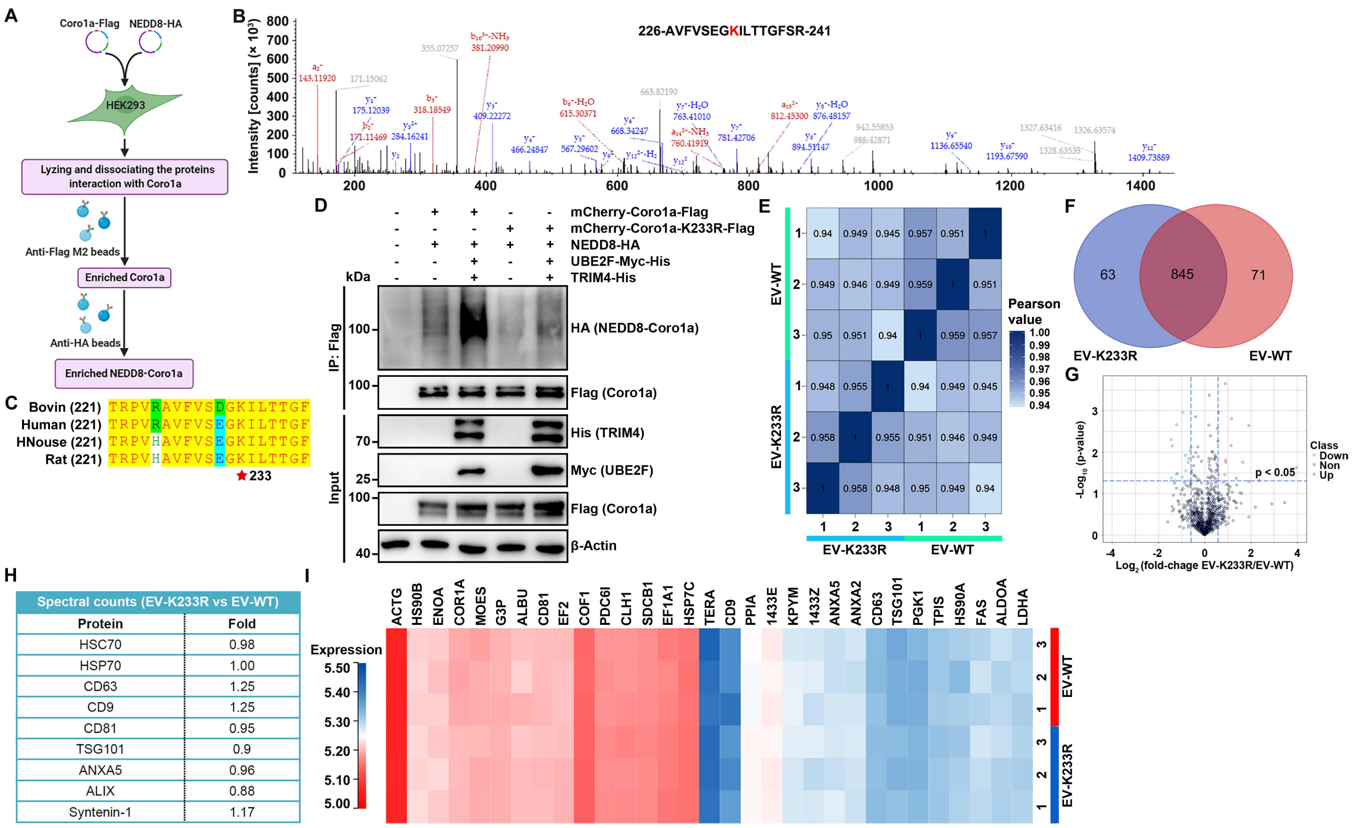

Supplementary Figure 5: Lysine 233 is the main neddylation site in Coro1a

(*A*) Schematics of NEDD8-Coro1a enrichment from HEK293 cells transfected with vectors for Coro1a-Flag and NEDD8-HA expression. (*B*) MS spectrum showing the lysine site that underwent neddylation in purified NEDD8-Coro1a. (*C*) Sequence alignment of Coro1a from the indicated species. The red asterisk indicates the conserved K233 site. (*D*) WB analysis of NEDD8-Coro1a and Coro1a in the lysates of HEK293 cells transfected with mCherry-Coro1a-Flag or mCherry-Coro1a-K233R-Flag and NEDD8-HA, UBE2F-Myc-His and TRIM4-His after IP with anti-Flag. (*E*) Sample correlation analysis of EV-WT and EV-K233R. (*F*) Venn diagram indicating the number of unique and overlapping proteins in EV-WT and EV-K233R. (*G*) Volcano plots showing quantitative differences in proteins in EV-WT and EV-K233R. Green and red dots indicate differences equal to or greater than 1.5-fold, while black dots indicate differences less than 1.5-fold. Dots above the dashed line indicate proteins for which the difference in expression was significant (false discovery rate [FDR] < 0.05). (*H*) Table showing the

fold-change in spectral counts for the indicated proteins obtained from proteomic profiling of pool fractions containing EV-WT and EV-K233R. (*I*) Heatmap of the 30 most commonly identified EV proteins from the ExoCarta exosome database and Coro1a from proteomic profiling of EV-WT and EV-K233R. The scale indicates intensity defined as $\Delta[\log_2(\text{value}+1)]$. Representative results from three independent experiments are shown.

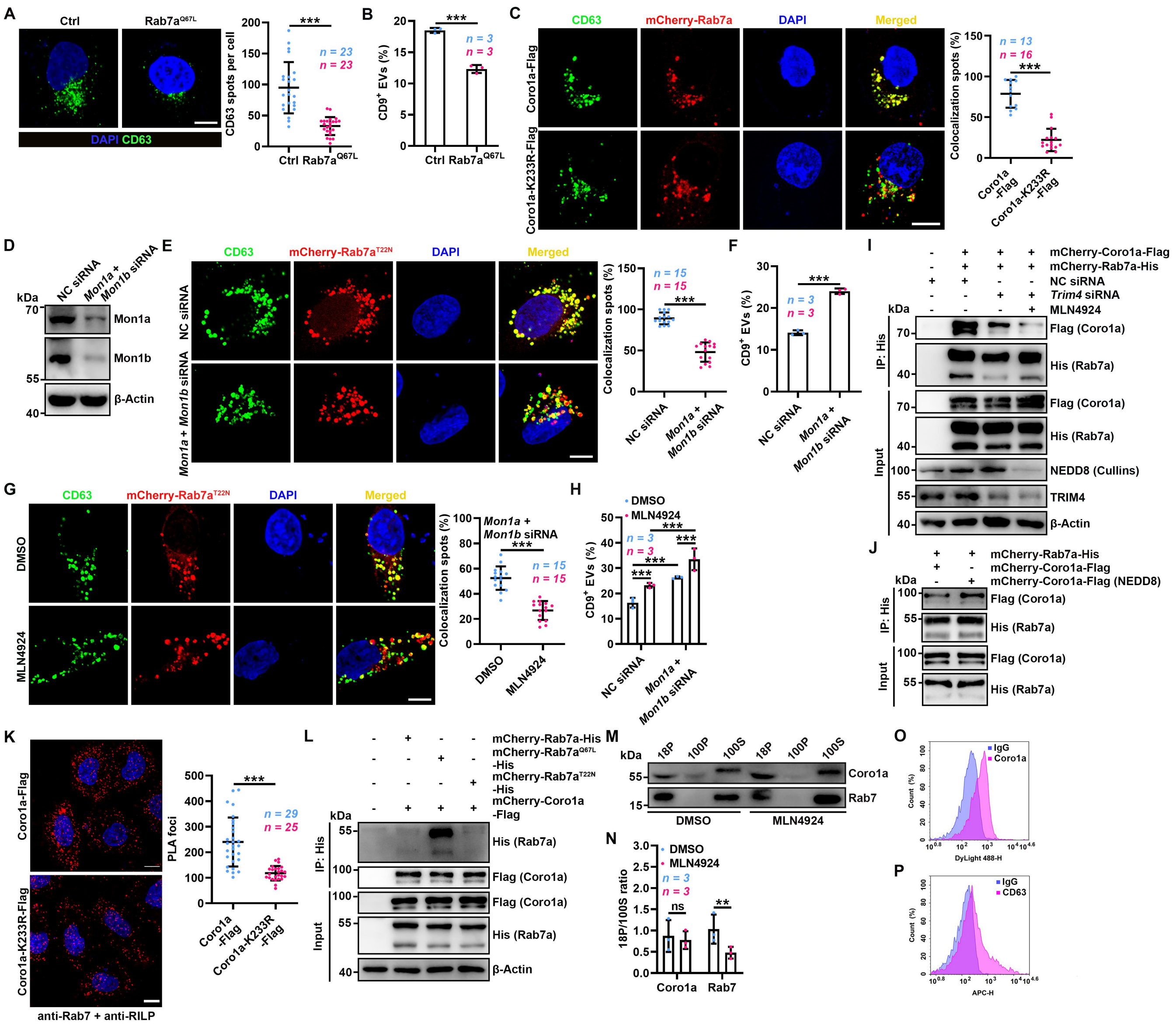

Supplementary Figure 6: NEDD8-Coro1a mediates the recruitment of Rab7 to MVBs

(*A*) Left, confocal microscopy analysis of the MVB marker CD63 in HeLa cells transfected with the constitutively active Rab7a^{Q67L} mutant. Scale bar, 10 μm. Right, quantification of CD63$^+$ spots per cell. Each dot indicates the number of CD63$^+$ spots per cell. (*B*) Flow cytometric ratio of CD9$^+$ EVs in the supernatants of HEK293 cells transfected with the constitutively active Rab7a^{Q67L} mutant. (*C*) Left, confocal microscopy analysis of CD63 and mCherry-Rab7a colocalization in HeLa cells transfected with mCherry-Rab7a and Coro1a-Flag or Coro1a-K233R-Flag vectors and then treated with 20 nM Baf A1 for 12 h. Scale bar, 10 μm. Right panel, each dot indicates the percentage of spots showing

colocalization per cell. (*D*) WB analysis of Mon1a and Mon1b in HEK293 cells transfected with NC or *Mon1a* and *Mon1b* siRNA. (*E*) Left, confocal microscopy analysis of CD63 and mCherry-Rab7a^{T22N} colocalization in HeLa cells transfected with mCherry-Rab7a vector and NC or *Mon1a* and *Mon1b* siRNA. Scale bar, 10 μm. Right, quantification of spots showing colocalization per cell. Each dot indicates the percentage of spots per cell showing colocalization. (*F*) Flow cytometric ratio of CD9^{+} EVs in the supernatants of HEK293 cells transfected with NC or *Mon1a* and *Mon1b* siRNA and then treated with DMSO or 100 nM MLN4924 for 12 h. (*G*) Left, confocal microscopy analysis of CD63 and mCherry-Rab7a^{T22N} colocalization in HeLa cells transfected with *Mon1a* and *Mon1b* siRNA along with mCherry-Rab7a^{T22N} vector and then treated with 100 nM MLN4924 for 12 h. Scale bar, 10 μm. Right, quantification of spots showing colocalization per cell. Each dot indicates the percentage of spots per cell showing colocalization. (*H*) Flow cytometric ratio of CD9^{+} EVs in the supernatants of HEK293 cells transfected with NC or *Mon1a* and *Mon1b* siRNA and then treated with 100 nM MLN4924 for 12 h. (*I*) WB analysis of Coro1a and Rab7a in the lysates of HEK293 cells transfected with vectors for mCherry-Coro1a-Flag and mCherry-Rab7a-His expression and NC or *Trim4* siRNA with or without 100 nM MLN4924 treatment for 12 h after IP with anti-His. (*J*) WB analysis of the *in vitro* interaction between the mCherry-Rab7a-His protein and mCherry-Coro1a-Flag protein from HEK293 cells transfected with mCherry-Coro1a-Flag and mock vectors or mCherry-Coro1a-Flag and NEDD8 vectors [mCherry-Coro1a-Flag (NEDD8)] after IP with anti-His. (*K*) Left, confocal microscopy analysis of PLA^{+} spots showing the interaction between Rab7 and RILP in HeLa cells overexpressing Coro1a-Flag or Coro1a-K233R-Flag. Scale bar, 10 μm. Right, quantification of PLA^{+} spots per cell. (*L*) WB analysis of Rab7a and Coro1a in the lysates of HEK293 cells transfected with vectors for mCherry-Coro1a-Flag and mCherry-Rab7a-His, mCherry-Rab7a^{Q67L}-His or mCherry-Rab7a^{T22N}-His expression after IP with

anti-Flag. (*M*, *N*) WB analysis (*M*) and the ratios (*N*) of mCoro1a to cytoplasmic Coro1a and mRab7 to cytoplasmic Rab7 in HEK293 cells treated with DMSO or 100 nM MLN4924 for 12 h. Postnuclear lysates were subjected to centrifugation at 18,000 × g for 20 min and then 2 h at 100,000 × g. 18P and 100P correspond to the pellets after the 18,000 × g and 100,000 × g spins, respectively. 100S corresponds to the supernatant after centrifugation at 100,000 × g. The ratio of the Coro1a and Rab7a signals in 18P and 100S was determined by gray value analysis. (*O*, *P*) Flow cytometric ratio of Coro1a$^+$ EVs from HEK293 cells captured with anti-CD63-coated latex beads (*O*) or CD63$^+$ EVs from HEK293 cells captured with anti-Coro1a-coated latex beads (*P*). Representative results from three independent experiments are shown. *n*, sample number; ns, not significant; $^{**}P < 0.01$ and $^{***}P < 0.001$ (unpaired two-tailed Student's *t*-test in *A*, *B*, *E-G*, *K*, *N*; unpaired Mann-Whitney test in *C*; one-way ANOVA followed by Tukey test in *H*; mean ± s.d.).

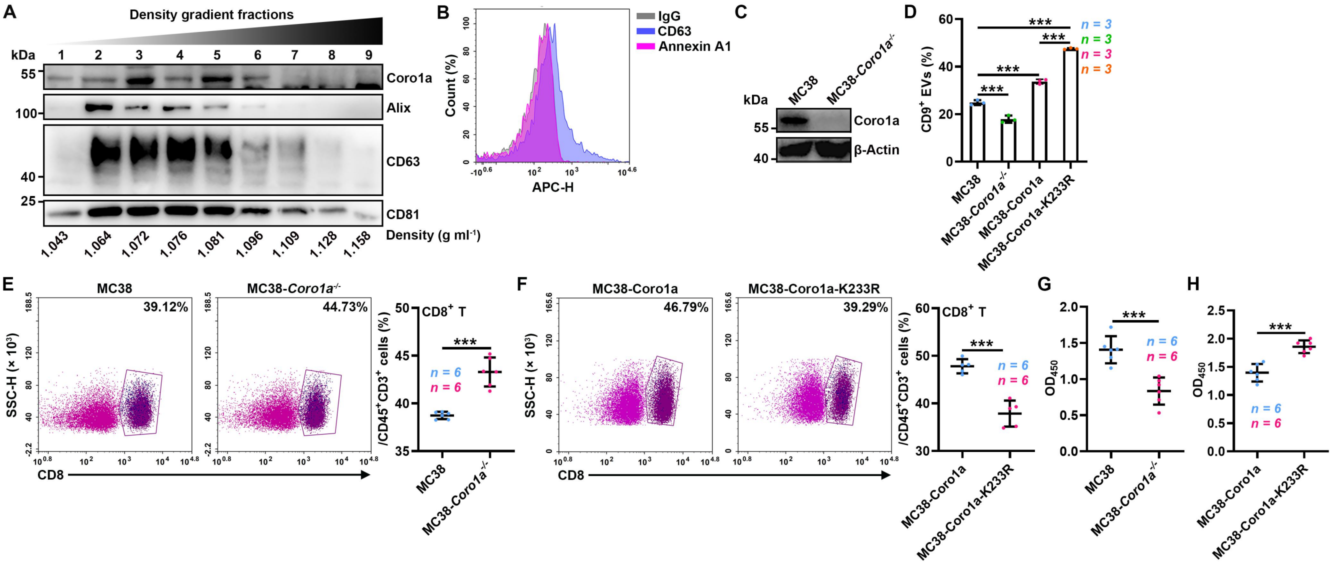

Supplementary Figure 7: Coro1a is an ideal target for the regulation of EV secretion

(*A*) WB analysis of Coro1a and the indicated EV markers in different iodixanol gradient fractions obtained from HEK293 cell supernatants. (*B*) Flow cytometric analysis of Annexin A1 and CD63 on EVs from HEK293 cells captured with anti-Coro1a-coated latex. (*C*) WB analysis of Coro1a in MC38-*Coro1a*$^{-/-}$ cells. (*D*) Flow cytometric ratio of CD9^{+} EVs in the supernatants of MC38 or MC38-*Coro1a*$^{-/-}$ cells or those of MC38-Coro1a or MC38-Coro1a-K233R cells. (*E*, *F*) Quantification of the percentage of CD45^{+}CD3^{+}CD8^{+} T cells in the dLNs from MC38 or MC38-*Coro1a*$^{-/-}$ tumor-bearing mice (*E*) or MC38-Coro1a or MC38-Coro1a-K233R (*F*) tumor-bearing mice on day 20 by flow cytometry. (*G*, *H*) ELISA of EV CD9 in the sera from MC38 or MC38-*Coro1a*$^{-/-}$ tumor-bearing mice (*G*) or MC38-Coro1a or MC38-Coro1a-K233R (*H*) tumor-bearing mice. Representative results from three independent experiments are shown. *n*, sample number; ****P* < 0.001 (unpaired two-tailed Student's *t*-test except for one-way ANOVA followed by Tukey test in *D*; mean ± s.d.).
