# Supplementary material for: Neddylation of Coro1a determines the fate of multivesicular bodies and biogenesis of extracellular vesicles
Source: J Extracell Vesicles. 2021 Oct 8;10(12):e12153. doi: 10.1002/jev2.12153 (PMC8500273; doi:10.1002/jev2.12153)
Supplement: Supplementary file 2 — Supporting Information [file JEV2-10-e12153-s005.docx]

**Supplementary Table 1**

| REAGENT or RESOURCE | SOURCE | IDENTIFIER |
| --- | --- | --- |
| Antibodies (applications) | | |
| anti-mouse CD9 PE | BioLegend | Cat: 124806 |
| anti-human CD9 PE | BioLegend | Cat: 312105 |
| anti-human CD63 APC | BioLegend | Cat: 353007 |
| anti-mouse CD81 APC | BioLegend | Cat: 104909 |
| anti-human CD81 APC | BioLegend | Cat: 349510 |
| anti-Annexin A1 APC | BioLegend | Cat: 831603 |
| Mouse IgG1, κ Isotype Ctrl (FC) Antibody APC | BioLegend | Cat: 400121 |
| Rat IgG2b, κ Isotype Ctrl Antibody PE | BioLegend | Cat: 400607, |
| Rat IgG2a, κ Isotype Ctrl Antibody PE | BioLegend | Cat: 400507 |
| Purified anti-mouse CD16/32 Antibody | BioLegend | Cat: 101301 |
| eBioscience^TM^ Fixable Viability Dye eFluor^TM^ 520 | Invitrogen | Cat: 65-0867-14 |
| anti-mouse CD45 PB | BioLegend | Cat: 103126 |
| anti-mouse CD3 APC | BioLegend | Cat: 100235 |
| anti-mouse CD8α PerCP | Invitrogen | Cat: 45-0081-82 |
| anti-mouse PD-1 PE | BioLegend | Cat: 135205 |
| anti-mouse TIM3 PE | BioLegend | Cat: 119703 |
| anti-mouse Granzyme B PE | Invitrogen | Cat: 12-8898-82 |
| anti-mouse Ki-67 PE | BioLegend | Cat: 151209 |
| Mouse anti-CD63 (IF) | Invitrogen | Cat: MA1-19281 |
| Rabbit anti-CD63 (WB) | Abclonal | Cat: A5271 |
| Mouse anti-Coro1a (IP) | Santa Cruz | Cat: sc-100925 |
| Rabbit anti-Coro1a (WB,FC,IF) | Abcam | Cat: ab228635 |
| Mouse anti-Rab7 (WB,IF) | Abcam | Cat: ab126712 |
| Rabbit anti-Rab7 (Cryo-EM) | Bioss | Cat: bs-6703R |
| Rabbit anti-EEA1 | Abcam | Cat: ab109110 |
| Rabbit anti-HRS | Abcam | Cat: ab155539 |
| Rabbit anti-LAMP1 | Abcam | Cat: ab24170 |
| Rabbit anti-GDI2 | Abclonal | Cat: A8615 |
| Rabbit anti-RILP | Proteintech | Cat: 13574-1-AP |
| Rabbit anti-CD81 | Affinity | Cat: DF2306 |
| Rabbit anti-Tsg101 | Abclonal | Cat: A1692 |
| Rabbit anti-Alix | Proteintech | Cat: 12422-1-AP |
| Rabbit anti-GM130 | Abcam | Cat: ab52649 |
| Rabbit anti-NEDD8 | Abcam | Cat: ab81264 |
| Mouse anti-Ubiquitin (P4D1) | CST | Cat: 3936 |
| Rabbit anti-Cullin3 | Sangon Biotech | Cat: D120492 |
| Rabbit anti-UBA3 | Abcam | Cat: ab124728 |
| Rabbit anti-UBE2F | Bioss | Cat: bs-8378R |
| Rabbit anti-UBE2M | Abcam | Cat: ab109507 |
| Rabbit anti-TRIM4 | Abclonal | Cat: A15922 |
| Rabbit anti-Mon1a | Abclonal | Cat: A17946 |
| Rabbit anti-Mon1b | Abclonal | Cat: A17940 |
| Rabbit anti-β-Actin | Abclonal | Cat: AC026 |
| Rabbit anti-Flag | CST | Cat: 14793 |
| Rabbit anti-HA | CST | Cat: 3724 |
| Rabbit anti-His | MBL | Cat: PM032 |
| Rabbit anti-Myc | Abmart | Cat: M20002 |
| Goat anti-mouse IgG HRP | MultiSciences | Cat: 70-GAM007 |
| Goat anti-rabbit IgG HRP | MultiSciences | Cat: 70-GAR007 |
| VeriBlot for IP Detection Reagent (HRP) | Abcam | Cat: 131366 |
| Mouse Control IgG | Abclonal | Cat: AC011 |
| Rabbit Control IgG | Abclonal | Cat: AC005 |
| Purified anti-mouse CD63 Antibody | BioLegend | Cat: 143901 |
| Ultra-LEAF™ Purified anti-human CD63 Antibody | BioLegend | Cat: 353039 |
| Biotin anti-mouse CD9 Antibody | BioLegend | Cat: 124803 |
| Biotin anti-mouse CD81 Antibody | BioLegend | Cat: 104903 |
| Biotin anti-mouse F4/80 Antibody | BioLegend | Cat: 123105 |
| Goat anti-rabbit IgG DyLight 488 | MultiSciences | Cat: 70-GAR4882 |
| Goat anti-mouse IgG DyLight 488 | MultiSciences | Cat: 70-GAM4882 |
| Goat anti-mouse IgG DyLight 594 | MultiSciences | Cat: 70-GAM5942 |
| Goat anti-rabbit IgG DyLight 594 | MultiSciences | Cat: 70-GAR5942 |
| Donkey Anti-Rabbit IgG H&L (Alexa Fluor^®^ 568) preadsorbed | Abcam | Cat: 175692 |
| **Transfection Reagents** | | |
| JetPEI | Polyplus | Cat: 101-10N |
| PEI | Polysciences | Cat: 23966-2 |
| INTERFERin^@^ | Polyplus | Cat: 409-10 |
| *Trans*IT-TKO | Mirus | Cat: MIR 2150 |
| Other Reagents | | |
| MLN4924 | Selleck | Cat: S7109 |
| Baf A1 | MCE | Cat: HY-100558 |
| Anti-FLAG^®^M2 Magnettic Beads | Sigma | Cat: M8823 |
| Anti-His-tag Magnettic Beads | MBL | Cat: D291-11 |
| Anti-HA-tag Magnettic Beads | MBL | Cat: M180-11 |
| Anti-c-Myc Magnettic Beads | Thermo | Cat: 88842 |
| Protein A/G PLUS-Agarose | Santa Cruz | Cat: sc2003 |
| 4-μm Aldehyde/Sulfate latex beads | Invitrogen | Cat: 1736853 |
| BCA Protein Assay Kit | Thermo | Cat: 23225 |
| Avidin-HRP | Biolegend | Cat:79004 |
| TMB ELISA Substrate Solution | Biolegend | Cat: 79057 |
| Duolink^®^ In Situ PLA^®^ Probe anti-Rabbit PLUS | Sigma | Cat: DUO92002 |
| Duolink^®^ In Situ PLA^®^ Probe anti-Mouse MINUS | Sigma | Cat: DUO92004 |
| Duolink^®^ In Situ Detection Reagents Red | Sigma | Cat: DUO92008 |
| Duolink^®^ In Situ Wash Buffers | Sigma | Cat: DUO82049 |
| Neddylation kit | Enzo | Cat: BML-UW0590 |
| UBE2F Fusion Protein | Proteintech | Cat: Ag10729 |
| eBioscience™ Factor Staining Buffer Set | Invitrogen | Cat: 00-5523-00 |
| PMA | Sigma | Cat: P1585 |
| Ionomycin | BioGems Internationa | Cat: 5608212 |
| FBS | HyClone | Cat: SV30160.03 |
| GM-CSF | Novoprotein | Cat: CJ46 |
| OptiPrep^TM^ (Iodixanol) | StemCell | Cat: 07820 |
| Fast Silver Stain Kit | Beyotime | Cat: P0017S |
| Coomassie brilliant blue R-250 | Solarbio | Cat: 6104-59-2 |
| Plasmids | | |
| plvx-mCherry-C1 | Clontech | Cat: 632561 |
| pcDNA3.1 | Invitrogen | Cat: V79520 |
| plv5-Cas9-Blast | Merck | Cat: CAS9BST-1EA |
| U6-gRNA: hPGK-puro-2A-tBFP | Merck | Cat: CRISPR18 |
| siRNA for gene knockdown | | |
| NC siRNA F: 5’-UUCUCCGAACGUGUCACGUdTdT-3’ | Genepharma | N/A |
| NC siRNA R: 5’-ACGUGACACGUUCGGAGAAdTdT-3’ | Genepharma | N/A |
| *hUbe2f* siRNA F: 5’-GAUGACUACAUCAAACGUUAUdTdT-3’ | Genepharma | N/A |
| *hUbe2f* siRNA R: 5’-AUAACGUUUGAUGUAGUCAUCdTdT-3’ | Genepharma | N/A |
| *hUbe2m* siRNA F: 5’-GCGGAUCCAGAAGGACAUAAAdTdT-3’ | Genepharma | N/A |
| *hUbe2m* siRNA R: 5’-UUUAUGUCCUUCUGGAUCCGCdTdT-3’ | Genepharma | N/A |
| *hTrim4* siRNA F: 5’-GCUGAAUGAGAACACGUUAAAdTdT-3’ | Genepharma | N/A |
| *hTrim4* siRNA R: 5’-UUUAACGUGUUCUCAUUCAGCdTdT-3’ | Genepharma | N/A |
| *hCoro1a* siRNA F: 5’-CUGUGCUGUCAACCCUAAGUUdTdT-3’ | Genepharma | N/A |
| *hCoro1a* siRNA R: 5’-AACUUAGGGUUGACAGCACAGdTdT-3’ | Genepharma | N/A |
| *hMon1a* siRNA F: 5’-GAAUGCCUUGAUGGCACAUUGdTdT-3’ | Genepharma | N/A |
| *hMon1a* siRNA R: 5’-CAAUGUGCCAUCAAGGCAUUCdTdT-3’ | Genepharma | N/A |
| *hMon1b* siRNA F: 5’-GGUCUGCCUCCUAGUUGAAUCdTdT-3’ | Genepharma | N/A |
| *hMon1b* siRNA R: 5’-GAUUCAACUAGGAGGCAGACCdTdT-3’ | Genepharma | N/A |
| gRNA for gene knockout | | |
| *mCoro1a* gRNA-1: 5’-ACCAGGCGATGTCTAGCACAGG-3’ | Merck | N/A |
| *mCoro1a* gRNA-2: 5’-GTAGACAAGAACGTGCCCCTGG-3’ | Merck | N/A |
| Primers for mice identification | | |
| *Lysm^cre^-*M-F: 5’-CCCAGAAATGCCAGATTACG-3’ | Cyagen Biosciences | N/A |
| *Lysm^cre^*-M-R: 5’-CTTGGGCTGCCAGAATTTCTC-3’ | Cyagen Biosciences | N/A |
| *Lysm^cre^*-WT-F: 5’-TTACAGTCGGCCAGGCTGAC-3’ | Cyagen Biosciences | N/A |
| *Ube2m^fl/fl^*-F: 5’-CCGTGTCGTGAAGATTGTGAAGG-3’ | GemPharmatech | N/A |
| *Ube2m^fl/fl^*-R: 5’-ACCTCCACTGTCCTTCCTCGTCTC-3’ | GemPharmatech | N/A |
| *Ube2f^fl/fl^*-F: 5’-CCAGGGTGGAAAATTTCAGTTT-3’ | GemPharmatech | N/A |
| *Ube2f^fl/fl^*-R1: 5’-GCGAGCTCAGACCATAACTTCG-3’ | GemPharmatech | N/A |
| *Ube2f^fl/fl^*-R2: 5’-CCCTGGAATTTCGGTATTATA-3’ | GemPharmatech | N/A |
| *Coro1a^fl/fl^*-F: 5'-AGAAGAGAAGAGGAAGTGGTTGTC-3' | Cyagen Biosciences | N/A |
| *Coro1a^fl/fl^*-R: 5'-AGGATGTGTCCTATAGATTTGGGC-3 | Cyagen Biosciences | N/A |

**CONTACT FOR REAGENT AND RESOURCE SHARING**

Further information and requests for resources and reagents should be directed to and will be fulfilled by the Lead Contact, Zhijian Cai ([caizj@zju.edu.cn](mailto:caizj@zju.edu.cn)).
